# Mutation in the Two-Component System Regulator YycH Leads to Daptomycin Tolerance in Methicillin-Resistant Staphylococcus aureus upon Evolution with a Population Bottleneck

**DOI:** 10.1128/spectrum.01687-22

**Published:** 2022-08-01

**Authors:** Jordy Evan Sulaiman, Long Wu, Henry Lam

**Affiliations:** a Department of Chemical and Biological Engineering, The Hong Kong University of Science & Technology, Kowloon, Hong Kong; University of Exeter

**Keywords:** MRSA, population bottleneck, daptomycin, evolution, tolerance, YycH, YycI, WalKR, two-component system

## Abstract

Adaptive laboratory evolution (ALE) is a useful tool to study the evolution of antibiotic tolerance in bacterial populations under diverse environmental conditions. The role of population bottlenecks in the evolution of tolerance has been investigated in Escherichia coli, but not in a more clinically relevant pathogen, methicillin-resistant Staphylococcus aureus (MRSA). In this study, we used ALE to evolve MRSA under repetitive daptomycin treatment and incorporated population bottlenecks following antibiotic exposure. We observed that the populations finally attained a tolerance mutation in the *yycH* gene after 2 weeks of evolution with population bottlenecks, and additional mutations in *yycI* and several other genes further increased the tolerance level. The tolerant populations also became resistant to another glycopeptide antibiotic, vancomycin. Through proteomics, we showed that *yycH* and *yycI* mutations led to the loss of function of the proteins and downregulated the WalKR two-component system and the downstream players, including the autolysin Atl and amidase Sle1, which are important for cell wall metabolism. Overall, our study offers new insights into the evolution of daptomycin tolerance under population bottlenecking conditions, which are commonly faced by pathogens during infection; the study also identified new mutations conferring daptomycin tolerance and revealed the proteome alterations in the evolved tolerant populations.

**IMPORTANCE** Although population bottlenecks are known to influence the evolutionary dynamics of microbial populations, how such bottlenecks affect the evolution of tolerance to antibiotics in a clinically relevant methicillin-resistant S. aureus (MRSA) pathogen are still unclear. Here, we performed *in vitro* evolution of MRSA under cyclic daptomycin treatment and applied population bottlenecks following the treatment. We showed that under these experimental conditions, MRSA populations finally attained mutations in *yycH*, *yycI*, and several other genes that led to daptomycin tolerance. The discovered *yycH* and *yycI* mutations caused early termination of the genes and loss of function of the proteins, and they subsequently downregulated the expression of proteins controlled by the WalKR two-component system, such as Atl and Sle1. In addition, we compared our proteomics data with multiple studies on distinct daptomycin-tolerant MRSA mutants to identify proteins with a consistent expression pattern that could serve as biological markers for daptomycin tolerance in MRSA.

## INTRODUCTION

Antibiotic tolerance is the ability of bacterial populations to survive, but not grow or replicate, under lethal doses of antibiotics. Persistence is a phenomenon where tolerance occurs only in a subpopulation of cells called persisters (1, 2), which are phenotypic variants with no genetic difference compared to the susceptible cells but are better positioned to withstand antibiotic challenges owing to differential expression of certain genes or proteins and/or shifted metabolism. Although persistence is regarded as a phenotypic trait (3, 4), it is known that persistence is highly evolvable. Populations repetitively treated with antibiotics quickly adapt to the treatment and exhibit an increased level of tolerance after merely a few cycles, by accommodating higher fractions of persisters than their progenitors (5). In some cases, the populations also become resistant after repetitive antibiotic treatments, such as when there are positive epistatic interactions between specific tolerance and resistance mutations resulting in a higher fitness (6, 7), or when the survival advantages of the resistant mutants exceed those of the tolerant mutants (8).

Adaptive laboratory evolution (ALE) is an emerging strategy to study the development of tolerance and explore evolutionary dynamics under diverse treatment conditions (8–16). With this experimental approach, it has been shown that several factors affect the rate of persistence and tolerance evolution, such as the treatment frequency (11) and the phase of growth of the cultures during treatment (8). In addition, treatments using different classes of antibiotics (9), different drug combinations (13), and different durations of exposure (10) yield a different set of tolerance mutations, highlighting the large mutational target size and the complexity of the tolerance phenotype. Recently, the role of population bottlenecks for the evolution of persistence and tolerance has been investigated (17). Population bottlenecks are sudden and severe reductions in population size which frequently occur during infection by numerous pathogens (18–21). Other bottlenecking events also happen postcolonization, such as host-parasite interactions and the intracellular uptake of bacteria or an attack by the immune system, all of which vigorously trim the size of bacterial populations (22, 23). It is known that bottlenecks influence the evolutionary dynamics of microbial populations mainly by increasing the impact of genetic drift and reducing the mutational supply rate. Briefly, as populations are forced through a small bottleneck, a random subpopulation of the cells is retained, largely independent of the fitness of the cells. A large fraction of the emerging mutants is lost, reducing the genetic diversity and hence the speed of evolution. At the same time, bottlenecks introduce more randomness to the evolution process, hence increasing the variability of the outcome of evolution. By studying the impact of population bottlenecks on the evolutionary dynamics of E. coli persistence, Windels et al. showed that the fitness landscape associated with persistence has a rugged topography, with distinct trajectories toward increased persistence that are accessible to evolving populations (17). By comparing different bottleneck sizes, Windels and colleagues concluded that smaller bottlenecks restrict the adaptive potential of populations and result in a more heterogeneous evolutionary outcome. Although that study contributed to a better understanding of how population bottlenecks guide the adaptation of E. coli to antibiotic treatment and revealed new genes involved in E. coli persistence, how the bottleneck affects the evolution of persistence and tolerance in other clinically relevant pathogens and antibiotics is still unclear.

In the present study, we employed ALE to investigate the evolution of MRSA under repetitive daptomycin treatment using a modified protocol that incorporated population bottlenecks following antibiotic exposure. Our work builds on the previous study by Windels et al. (17) that is now being extended to study how population bottlenecks affect the evolution of persistence and tolerance in MRSA. We observed that although tolerance development was slower when population bottlenecks were applied, the populations finally attained tolerance mutation in the *yycH* gene after 2 weeks of treatment. Furthermore, additional mutations in *yycI* and several other genes led to an even higher tolerance level. The two accessory proteins YycH and YycI control the WalKR two-component system (TCS) and have been extensively studied in Bacillus subtilis (24). However, they have only been recently explored in S. aureus, and much less is known regarding their functional role. The WalKR TCS is the only one of a total of 16 sets of TCSs in S. aureus that is essential for bacterial growth, since it maintains cell wall metabolism by controlling the expression of cell wall-lysing enzymes (25–27). Among the genes controlled by the WalKR system in S. aureus, several of them are peptidoglycan hydrolases, including the muramidase IsaA and SceD, endopeptidase LytM, CHAP domain autolysin SsaA, and amidase Sle1, as well as the major autolysin AtlA, which has glucosaminidase and amidase activities (26). Changes in the expression of the WalKR TCS have been shown to cause resistance to the last-resort antibiotic, vancomycin, by preventing it from reaching its target molecule through cell wall thickening and reduced cross-linking of the peptidoglycan (28–30). Indeed, this vancomycin resistance phenotype was also observed in our daptomycin-tolerant evolved populations bearing a mutation in *yycH*. Recent studies have shown that *walKR* mutations commonly lead to cross-resistance to lipopeptides (e.g., daptomycin), glycopeptides (e.g., vancomycin), and lipoglycopeptides (e.g., dalbavancin) that target cell wall biosynthesis (31–33).

Omics methodology has been recently applied to study the adaptation mechanisms of evolved tolerant and resistant strains from such *in vitro* evolution experiments (4), both in E. coli (12, 34) and S. aureus (13, 35–37). Here, we performed quantitative proteomics and compared the proteome profiles of the evolved populations to that of the ancestral strain to gain mechanistic insights into their adaptation.

## RESULTS AND DISCUSSION

### ALE using a population bottleneck generated daptomycin-tolerant populations bearing mutations in *yycH* and other genes.

**FIG 1:**
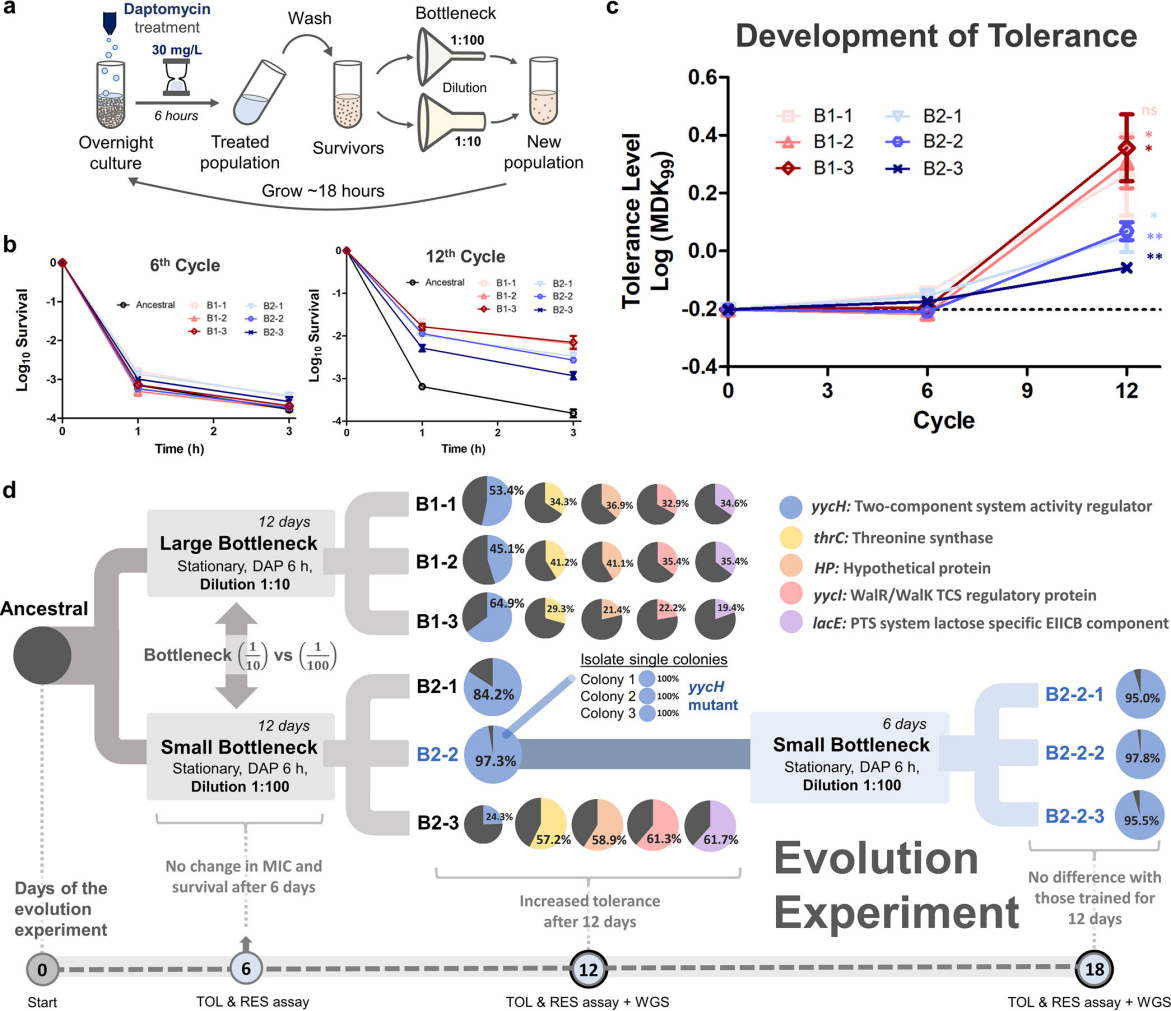
Laboratory evolution using population bottleneck-generated daptomycin-tolerant populations bearing a mutation in the *yycH* gene. (a) Experimental protocol used during the evolution experiment. Stationary-phase MRSA was repetitively treated with daptomycin (30 mg/liter) for 6 h. The survivors were subjected to a bottleneck (either a large bottleneck, 1:10 dilution, or small bottleneck, 1:100 dilution), subsequently regrown overnight, and the cycle was repeated. Three parallel populations were used for each experimental protocol. (b) Time-kill curves of the ancestral and evolved populations after 6 (left) and 12 cycles (right) of evolution upon daptomycin treatment (10 mg/liter) for 3 h (mean ± standard error of the mean [SEM], *n* = 3). (c) MDK_99_ (minimum duration for killing 99% of the population) measurements of the evolved populations after 6 and 12 cycles of evolution (mean ± SEM, *n* = 3). The horizontal dashed line shows the mean MDK_99_ of the ancestral population. Significance of difference with the ancestral: *, Bonferroni-adjusted *P < *0.05; **, Bonferroni-adjusted *P < *0.01; ns, not significant (two-tailed *t* test on log-transformed values, followed by Bonferroni correction). (d) Summary of the evolution experiment. Three parallel MRSA populations were evolved with large bottlenecks (B1-1, B1-2, and B1-3) and three were evolved with small bottlenecks (B2-1, B2-2, and B2-3). After 12 cycles, the populations were subjected to tolerance and resistance assays and whole-genome sequencing. B2-1 and B2-2 harbored a mutation only in the *yycH* gene, whereas B1-1, B1-2, B1-3, and B2-3 harbored mutations in *yycH*, *thrC*, *yycI*, *lacE*, and a hypothetical protein (*hp*) gene (for details of the mutation, see Table S2 and Table S3 in the supplemental material). The evolution experiment was prolonged for 6 cycles on B2-2 population using small bottlenecks, generating B2-2-1, B2-2-2, and B2-2-3, which all bore a mutation in the *yycH* gene. Pie charts show the proportion of specific mutations in the population based on the number of alternate reads divided by the total number of reads at the locus. Black color indicates the percentage of reference reads, whereas blue, red, yellow, purple, and orange colors indicate the percentage of alternate reads for the *yycH*, *yycI*, *thrC*, *lacE*, and *hp* genes.

To see the effect of population bottlenecks on the evolution of tolerance in MRSA under daptomycin treatment, we subjected the cells to ALE, where the populations were forced through either a large bottleneck (1:10) or small bottleneck (1:100) following antibiotic treatment that enriched for persisters or tolerant cells, with three parallel populations for each experimental condition (Fig. 1a and d). To simulate random population loss without introducing selective pressure during the bottlenecking, a subset of the population that survived antibiotic treatment was randomly picked to proliferate simply by diluting the population. After 6 days of the evolution experiment, we observed no change in the MIC or the tolerance level of all 6 populations in both the large bottleneck (B1-1, B1-2, B1-3) and small bottleneck (B2-1, B2-2, B2-3) treatment conditions (Fig. 1b and c). After 12 days, all 6 populations from the large and small bottlenecks became tolerant, although the tolerance level of the populations that evolved under the small bottleneck treatment was lower than that under the large bottleneck treatment (Fig. 1b and c). In addition, the MICs of the evolved populations toward daptomycin after 12 days of treatment were the same as those for the ancestral strain (see Table S1 in the supplemental material), and therefore the increased survival could not be attributed to resistance. While in this study we observed that tolerance evolved somewhere between 6 and 12 days of treatment, in a previous study when we performed an ALE experiment with the same protocol but without population bottlenecking, daptomycin tolerance developed after only a week of treatment (8).

To uncover the genetic profile governing the observed tolerance phenotypes, we subjected the evolved populations from the large and small bottlenecks to whole-genome sequencing. Table S2 and Table S3 summarize the mutations detected in the evolved populations. We observed that all 3 populations from the large bottleneck had the same set of mutations in *yycH*, *yycI*, *thrC*, *lacE*, and a hypothetical protein (*hp*) gene, whereas only one population from the small bottleneck had this set of mutations (B2-3). The two other populations from the small bottleneck, B2-1 and B2-2, had only one mutation in the *yycH* gene. The genes *yycH* and *yycI* are regulators of the bacterial TCS, which allows them to sense external stimuli and make necessary protective responses to cell wall defects and cell wall-active antibiotics. This system has been linked to daptomycin tolerance and resistance (38–43). Although *yycH* and *yycI* have been extensively studied in B. subtilis (24, 44–46), their role in the activation of the WalKR essential two-component system in S. aureus has only recently been explored. Several studies pointed to its function in regulating the production of autolysins (47) and controlling the cells’ susceptibility to vancomycin (48–50). Here, we also observed that the tolerant populations bearing only the *yycH* mutation (B2-1 and B2-2) and tolerant populations bearing mutations in *yycH*, *yycI*, *thrC*, *lacE*, and *hp* genes (B1-1, B1-2, B1-3, and B2-3) were resistant to vancomycin, with a 2.5-fold increase in the MIC compared to the ancestral population (see Table S1). As for the other mutated genes, *thrC* is involved in threonine biosynthesis, and *lacE* plays a role in the phosphoenolpyruvate-dependent sugar phosphotransferase system (PTS), which catalyzes the phosphorylation of incoming sugar substrates concomitantly with their translocation across the cell membrane. It must be noted that while the mutation in *thrC* and *hp* are single point mutations that commonly lead to an amino acid substitution, mutations in *yycH*, *yycI*, and *lacE* are deletions of several base pairs that could lead to more drastic changes in the protein sequence. The expected effects of a deletion in the *yycH*, *yycI*, and *lacE* genes on their protein sequence are summarized in Fig. S1.

Altogether, our results suggest that mutation in *yycH* led to daptomycin tolerance (B2-1 and B2-2) (Fig. 1b to d), and the addition of the other 4 mutations on top of the *yycH* mutation further increased the tolerance level of the population (B1-1, B1-2, B1-3). The reason why B2-3 has a lower tolerance level despite having the *yycH* mutation and the other 4 mutations might be because the proportion of cells with the *yycH* mutation is lower in the population (24%) compared to those in the other populations (between 45% and 97%). This may also be attributed to the smaller bottleneck, which is expected to slow the selective enrichment of the more tolerant strain(s). In addition, the 4 mutations (*yycI*, *thrC*, *lacE*, *hp* genes) seemed to always have a similar abundance within a population and always appeared together across different populations (B1-1, B1-2, B1-3, B2-3), and therefore they might be occurring in the same genetic background. This observation is particularly intriguing since the simultaneous occurrence of the 4 mutations in the same genetic background over multiple populations seems to be a rare event unless the genes are linked, and this finding therefore warrants further investigation. On the other hand, the mutation in *yycH* occurs at different frequencies compared to the other 4 mutations in different populations and therefore might be located in a different genetic background than the 4 mutations. In fact, comparing across populations, the abundance of *yycH* seems to move in the opposite direction as those of the *yycI thrC lacE hp* quadruple mutants, indicating a possible fitness competition between a *yycH* mutant and a *yycI thrC lacE hp* quadruple mutant. Nonetheless, this remains speculative, given the imprecise estimation of mutation abundance by population-wise whole-genome sequencing.

To see whether the extension of the evolution experiment would lead to more new mutations, we subjected B2-2 (containing ~97% *yycH* mutant) to another week of the evolution experiment. Prolonging the evolution using the same protocol with a small population bottleneck did not alter the genetic profile of the populations (B2-2-1, B2-2-2, and B2-2-3), and the abundance of the *yycH* mutation within the populations remained similar compared to those before the evolution (~95% to 98%) (Fig. 1d; see also Table S3).

### Treating tolerant populations in the exponential phase without population bottlenecks led to the emergence of resistance.

In a previous study, we showed that certain tolerant mutants with lower survival advantages could be outcompeted and invaded by daptomycin-resistant mutants bearing a mutation in the *mprF* gene under certain treatment conditions (8). To see whether the *yycH* tolerant mutant would be invaded by resistant mutants, we adopted the same treatment protocol as used in our previous study (8) and subjected B2-2 (containing ~97% *yycH* mutant) to another week of the evolution experiment. Rather than treating cells during the stationary phase with 30 mg/liter daptomycin for 6 h, we treated the cells during the exponential phase with a lower dose of daptomycin (10 mg/liter) for 1 h without bottlenecking (see Fig. S2a). Performing this evolution experiment on B2-2 without population bottlenecks for a week led to the addition of the previously observed *yycI thrC lacE hp* quadruple mutations (EXP1 and EXP3), similar to what we found in the large bottleneck condition (B1-1, B1-2, and B1-3). Interestingly, EXP3 also lost the *yycH* tolerance mutation and gained the well-known resistance mutation in the *mprF* gene. This might be because the survival advantage of the *yycH* tolerant mutant was lower than the *mprF* resistant mutant, thereby allowing the resistant mutant to invade the population, similar to what we have previously observed with some other tolerant mutants (8). Indeed, the MIC for EXP3 of daptomycin was increased 2-fold, indicating that the population had increased resistance (see Fig. S2a and Table S1). The mutation in the *mprF* gene was a deletion of 6 bp, leading to the substitution of 3 amino acids (IVY) with 1 amino acid (N) without changing the rest of the protein sequence (see Fig. S1).

### Loss of expression of YycH and YycI proteins in the evolved populations.

**FIG 2:**
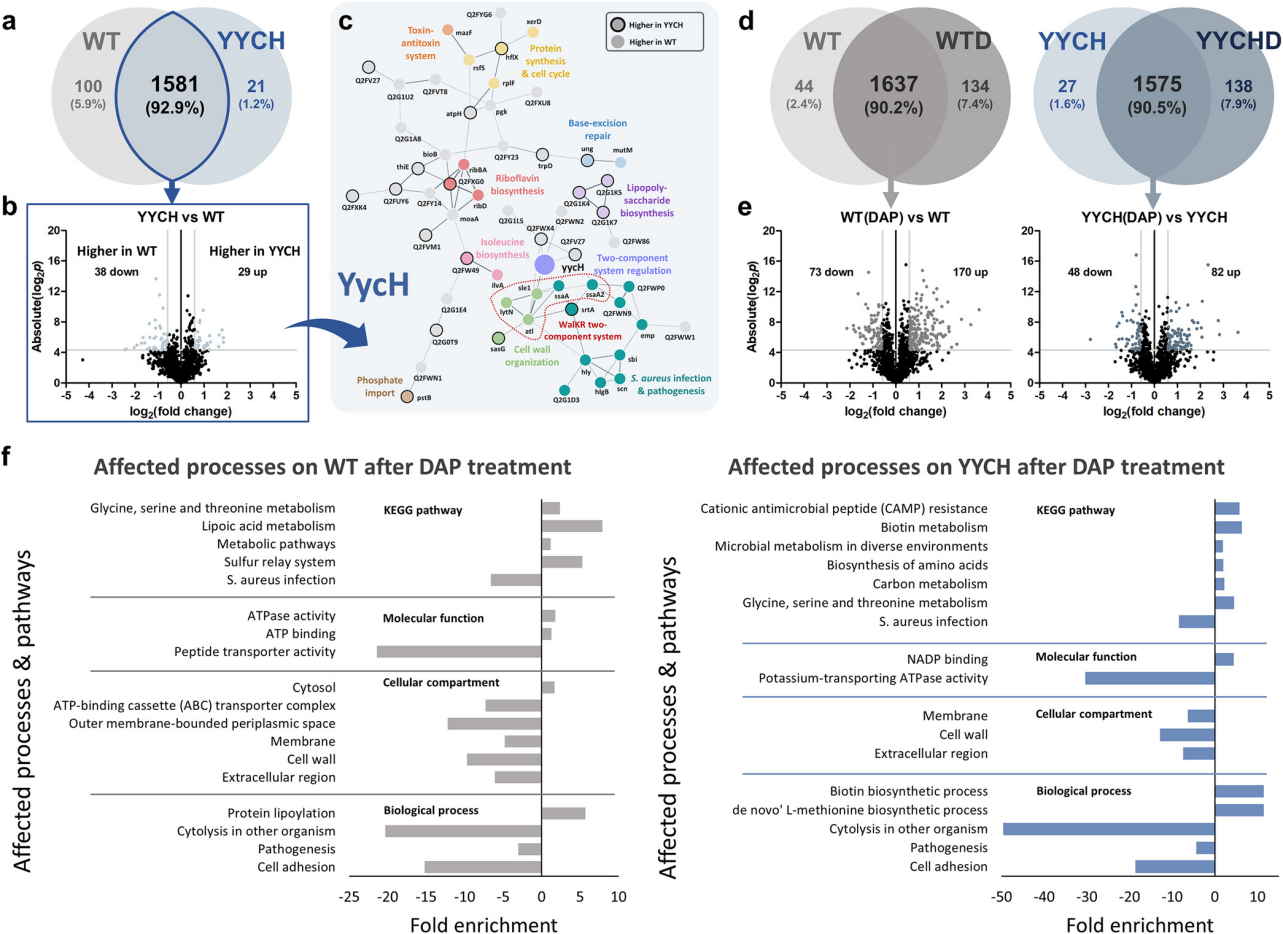
Proteomics analysis of the tolerant population. (a) Venn diagram for proteome comparison of the B2-2 tolerant (YycH) to the ancestral population in the absence of antibiotics. (b) Volcano plot of the B2-2 tolerant (YycH) population compared to the ancestral in the absence of antibiotics. Differentially expressed proteins (DEPs) were those with *P* values of <0.05 and an absolute fold change of >1.5, corresponding to the colored dots. (c) Protein-protein interaction network of the DEPs of the B2-2 tolerant population compared to the ancestral population, as predicted by STRING v11.5. The lines represent protein interactions (thicker lines indicate higher confidence), and the dots in different colors represent different protein functions. Nodes without function enrichment are colored gray. Nodes with black outlines are proteins with higher expression in the tolerant population, and nodes with no outlines are proteins with higher expression in the ancestral population. Node corresponding to the mutated gene in the tolerant population (*yycH*) is shown with twice the size of the other nodes. Nodes that belong to the WalKR two-component system are marked. (d) Venn diagrams for proteome comparison of the ancestral and B2-2 tolerant population upon daptomycin treatment (WTD and YYCHD) versus those before treatment (WT and YYCH). (e) Volcano plots of the ancestral and B2-2 tolerant population upon daptomycin treatment, compared to those before treatment. (f) GO analysis and pathway enrichment study (KEGG) by use of DAVID of the DEPs of the ancestral and B2-2 tolerant population after daptomycin treatment, compared to those before treatment. Fold enrichment is defined as the ratio of the proportion of the input information to the background information. Negative values indicate downregulation, and positive values indicate upregulation.

Next, we performed proteomic analysis to compare the proteome profile of the daptomycin-tolerant population (B2-2) to the ancestral strain, which would reveal the altered processes that underlie the tolerance phenotype. Combining all replicates, 1,681 and 1,602 distinct proteins were identified for the ancestral population and *yycH* tolerant population, respectively (Fig. 2a). In addition, we also treated each of the ancestral and tolerant populations with daptomycin and subjected them to proteomics analysis to gain insights into their adaptation strategy upon antibiotic treatment. The number of proteins identified from the treatment group was a bit higher than in the untreated group (1,771 and 1,713 for treated ancestral and tolerant populations, respectively) (Fig. 2d). Figure 2b and e show the volcano plots of fold changes against *P* values (from a two-tailed *t* test), highlighting the proteins of the tolerant population with different expression levels compared to the ancestral strain (Fig. 2b) and also the differentially expressed proteins in the ancestral and tolerant populations after daptomycin treatment (Fig. 2e). The list of differentially expressed proteins (DEPs) in comparing the tolerant population to the ancestral one is available in Table S4, whereas the list of DEPs from comparing the daptomycin-treated ancestral and tolerant populations to the untreated ones are available in Table S5 and Table S6, respectively. In addition, we also compared the proteome of the daptomycin-resistant population (EXP3) to that of the ancestral strain (see Fig. S2b), and through protein quantification, we highlighted the proteins of the resistant population with different expression levels compared to the ancestral strain (see Fig. S2c and d). The list of DEPs for comparing the resistant population to the ancestral is available in Table S7, whereas the list of DEPs for comparing the daptomycin-treated resistant population to the untreated one is available in Table S8.

Through proteomics, we discovered that the mutation of *yycH* in the tolerant population led to the loss of expression of the protein. While the average peptide spectrum matches (PSMs) for YycH in the ancestral strain were 18, 15, and 19 for the three biological replicates (average, ~17.33), it was 0 for all three biological replicates of the tolerant population (see Table S4). Similarly, the *yycI* mutation in the resistant population also led to the loss of expression of the protein, with average PSMs of 7, 9, and 15 for the three biological replicates (average, ~10.33), whereas it was 0 for all three biological replicates of the resistant population (see Table S7). Altogether, these findings indicated that the early termination of YycH and YycI proteins due to the 1-bp deletion is highly likely to abolish the protein function (see Table S2 and Fig. S1). It has been previously reported that both YycH and YycI stimulate WalK activity and are needed for full activation of the WalK kinase. Both of them are important in the direct regulation of WalKR-dependent cell wall metabolism, and the depletion of YycH and YycI levels has been shown to impair autolysis and increase wall teichoic acid (WTA) content of S. aureus cell walls (47). The reduced WalKR activation due to the deletion of *yycH* and *yycI* genes caused impaired cell wall turnover and ultimately reduced vancomycin efficacy (48). Indeed, both our tolerant and resistant populations with *yycH* and *yycI* mutations displayed increased MICs of vancomycin (see Table S1). Since daptomycin is also known to indirectly affect the S. aureus cell wall as part of its activity and changes in cell wall properties commonly lead to daptomycin tolerance or resistance (13, 37, 51–54), these impaired functions of YycH and YycI, which downregulated WalKR activity, might influence the cells’ susceptibility to daptomycin, similar to results with vancomycin. Unlike YycI, the other mutated protein, ThrC, was still detected in the resistant population, although the expression was 3.5-fold lower than in the ancestral strain (see Table S7). The reason why the protein was still expressed is probably that the mutation in *thrC* was a single point mutation (Cys36Tyr) rather than deletions like those that occurred in *yycH* and *yycI* (see Table S2). The proteins of the other mutated genes in the resistant strain were not detected in our proteomics analysis, which might have been due to their low abundance.

### Proteome alterations of the daptomycin-tolerant and -resistant populations.

We noticed that several processes were altered in the tolerant population, including proteins for protein synthesis and cell cycle, riboflavin biosynthesis, and isoleucine biosynthesis. The most evident change was the lower expression of proteins for S. aureus infection and pathogenesis (Fig. 2c), such as staphylococcal secretory antigen SsaA and SsaA2, uncharacterized leucocidin-like protein 1 (Q2FWP0) and 2 (Q2FWN9), extracellular matrix protein-binding protein Emp, immunoglobulin-binding protein Sbi, alpha-hemolysin protein Hly and gamma-hemolysin component B (HlgB), and staphylococcal complement inhibitor Scn. In addition, several proteins involved in cell wall metabolism were expressed at lower levels than in the ancestral strain, such as autolysin Atl, cell wall hydrolase LytN, and amidase Sle1. The expression of autolysin Atl and LytM was also lower in the resistant population (see Fig. S2d and Table S7). Our findings are consistent with a previous report showing *yycH* and *yycI* deletion mutants lead to the downregulation of the WalKR regulon through lower expression of cell wall hydrolase genes such as *atl* and *sle1*, resulting in an impaired autolytic phenotype (48). Here, we observed that most of the proteins whose expression is controlled by the WalKR system, such as Atl, Sle1, LytM, SsaA, and SsaA2 (47), were downregulated in the tolerant and resistant populations with the *yycH* and *yycI* mutations. In summary, our results suggest a mechanistic explanation for the increased tolerance of the mutants: the mutations in the *yycH* and *yycI* genes lead to the downregulation of the WalKR TCS, which subsequently affects the cell wall properties of the cells and their susceptibility to daptomycin.

Interestingly, one of the downregulated proteins in the daptomycin-tolerant population, immunoglobulin-binding protein Sbi (reduced by 2.3-fold) (see Table S4), has also been found downregulated in 5 different daptomycin-tolerant strains in previous studies bearing single point mutations in different genes conferring different levels of tolerance (two strains in one study [13] and three strains in another [37]), but not in daptomycin-resistant strains. Sbi is anchored to the cell envelope through binding to the lipoteichoic acid (LTA) and it plays a role in S. aureus virulence by inhibiting both the innate and adaptive immune responses, although its actual function in antibiotic tolerance or resistance is still unknown. Since an S. aureus LTA-defective mutant had reduced Sbi levels (55), it is possible that daptomycin-tolerant populations, in general, have a reduced number of LTA molecules anchored in the cell wall. Including our findings, the protein Sbi has the same trend of lower expression in 6 different daptomycin-tolerant strains and might be a marker for daptomycin tolerance in MRSA.

For the resistant population, aside from the differential expression of proteins for biosynthesis of amino acids, such as threonine, tryptophan, and arginine, they also have lower expression of many transporter proteins, which may play a role in their resistance phenotype (see Fig. S2d). One of the proteins that was expressed at much higher levels in the resistant population compared to the ancestral strain was the lactamase B domain-containing protein (see Table S7), which has sequence that is homologous to β-lactamases, enzymes that confer resistance to β-lactams. The higher expression level of this protein was also observed in another daptomycin-resistant strain bearing a mutation in a different location on the *mprF* gene (13).

### Affected processes and pathways following daptomycin treatment.

Through gene ontology (GO) analysis, we observed that the ancestral population downregulated proteins involved in cytolysis, S. aureus infection, pathogenesis, and cell adhesion following daptomycin treatment, suggesting that the lower expression of proteins for these processes is an adaptation for the cells to withstand daptomycin exposure (Fig. 2f). Indeed, the *yycH* tolerant population already had lower expression of proteins involved in S. aureus infection and pathogenesis compared to the ancestral strain, even in the absence of antibiotics (Fig. 2c), which is perhaps one of the adaptation strategies employed by the tolerant population toward daptomycin. The cationic antimicrobial peptide (CAMP) resistance pathway was upregulated in the tolerant population upon daptomycin treatment (Fig. 2f), which included proteins like the ABC transporter ATP-binding protein HrtA, the protein DltD, which plays a role in modulating cell wall properties, and staphylokinase. Another notable upregulated process and pathway in the tolerant population was biotin biosynthesis and metabolism.

The resistant population had many fewer changes in the proteome following daptomycin treatment (see Table S8), with only 83 DEPs, compared to 318 and 187 in the ancestral and tolerant populations, respectively. This might have been because they were already well-adapted to the antibiotic and could grow at higher drug concentrations. In the absence of daptomycin, the resistant population already exhibited some alterations in terms of proteome profile compared to the ancestral strain (see Fig. S2d), and perhaps this differential expression of proteins and alterations of specific processes are essential for their adaptation to daptomycin and are already sufficient for them to survive daptomycin treatment. For instance, the ancestral population that survived daptomycin treatment downregulated the expression of ABC transporters (Fig. 2f). However, the expression of ABC transporters was already lower in the resistant population than in the ancestral strain, even before daptomycin treatment (see Fig. S2d), indicating that the resistant population had become better adapted to the antibiotic.

### Conclusions.

Overall, we have shown that adaptive laboratory evolution in MRSA by using daptomycin with population bottlenecks generated daptomycin-tolerant mutants bearing mutations in the *yycH* two-component system regulator and several other genes. Complementing previous reports, our study revealed that mutations in *yycH* and *yycI* from laboratory evolution under bottlenecking conditions led to the loss of function of proteins and downregulation of the WalKR two-component system and its downstream players, such as autolysin Atl, amidase Sle1, and secretory antigens SsaA and SsaA2. The disruption of the cell wall maintenance function of these proteins might be the direct cause of the observed daptomycin tolerance, since the tolerant population also exhibited cross-resistance to another glycopeptide, vancomycin. Finally, we also highlighted the protein Sbi, which has been shown to be consistently expressed at lower levels in multiple daptomycin-tolerant strains across different studies and might serve as an important marker for daptomycin tolerance in MRSA.

## MATERIALS AND METHODS

### Bacterial strains and growth conditions.

The bacterial strain used in this study is methicillin-resistant S. aureus ATCC 43300. Exponential-phase cultures for evolution experiments and tolerance assays were prepared by incubating a 1:200 diluted overnight culture in cation-adjusted Mueller-Hinton (MH) broth until the optical density at 600 nm (OD_600_) reached ~0.1 at 37°C with shaking at 250 rpm. The MH broth used in this study was supplemented with Ca^2+^ to a final concentration of 50 mg/liter to mimic the physiological levels of calcium ions, which is important for the concentration-dependent bactericidal activity of daptomycin (56, 57). MH agar was used for colony counts.

### Evolution experiment.

The summary of the evolution experiment protocol is shown in Fig. 1a. Stationary-phase MRSA was repetitively treated with daptomycin (30 mg/liter) for 6 h to select for persisters, washed, subjected to a population bottleneck with either a 1:10 dilution (large bottleneck) or 1:100 dilution (small bottleneck), and regrown overnight for ~18 h. This evolution protocol (without population bottlenecks) was adopted from our previous study (8). Three independent populations were used for each evolution experiment protocol.

The summary of the overall evolution experiment scheme is shown in Fig. 1d. At the beginning of the evolution experiment, three parallel populations of MRSA were subjected to the slow evolution protocol with either a large bottleneck (B1-1, B1-2, B1-3) or a small bottleneck (B2-1, B2-2, B2-3). Tolerance and resistance assays were performed every 6 cycles to check for changes in phenotypes. After 12 cycles, the three populations were subjected to whole-genome sequencing to identify mutations associated with the altered phenotypes. The evolution experiment was prolonged for another 6 cycles on the B2-2 population by subjecting 3 parallel populations to the same evolution protocol with a small bottleneck (B2-2-1, B2-2-2, B2-2-3). At the end of the 18th cycle, all evolved populations were subjected to tolerance and resistance assays and whole-genome sequencing.

For the follow-up evolution experiment (see Fig. S2), the evolution experiment was performed for another 6 cycles on the B2-2 population by subjecting 3 parallel populations to a modified evolution protocol. Exponential-phase MRSA culture (prepared by incubating a 1:200 diluted overnight culture) was repetitively treated with daptomycin (10 mg/liter) for 1 h to select for persisters, washed, and regrown overnight for ~18 h.

### Tolerance and resistance assays.

The concentration of daptomycin used was 10 mg/liter for treatment of exponential-phase cells and 30 mg/liter for stationary-phase cells. The concentration of daptomycin used here was chosen to be similar to those from previous studies (58, 59), with the consideration that daptomycin exhibited a concentration-dependent bactericidal activity against high-inoculum stationary-phase cells (8, 60). To assess cell viability after antibiotic treatment, the numbers of survivors were counted by serially diluting cultures in MH broth and plating 100 μL on MH agar and spread plates. The MDK_99_ (minimum duration for killing 99% of the population) values, which represent the tolerance level, were extracted from the time-kill curves of the populations.

The MICs of the population were determined by the broth macrodilution method to spot for resistant variants. The MIC was determined by incubating ~5 × 10^5^ exponential-phase bacteria in MH medium overnight with various concentrations of antibiotics. The MIC value was determined as the lowest concentration without growth, according to EUCAST guidelines (8).

### Genomic extraction and whole-genome sequencing.

Genomic DNA from the ancestral and evolved populations was extracted using a DNeasy blood and tissue kit (Qiagen) according to the manufacturer’s protocol, with the following lysis buffer: 200 μg/mL lysostaphin solution in 20 mM Tris-HCl (pH 8.0), 2 mM sodium EDTA, and 1.2% Triton X-100. DNA was detected by agarose gel electrophoresis and quantified with a NanoVue Plus spectrophotometer (GE Healthcare). A genomic DNA sample of each population was sent to Groken Bioscience Ltd. for paired-end Illumina sequencing at 2 × 150-bp read length and 350-bp insert size, similar to a previous study (9). A total amount of 1 μg of DNA per sample was used as input material for the DNA sample preparation. Sequencing libraries were generated using a NEBNext Ultra DNA library prep kit for Illumina (NEB, USA) following the manufacturer’s protocol, and index codes were added to attribute sequences to each sample. Briefly, the DNA sample was fragmented by sonication to a size of 350 bp, and then DNA fragments were end-polished, A-tailed, and ligated with the full-length adaptor for Illumina sequencing with further PCR amplification. Finally, PCR products were purified (AMPure XP system) and libraries were analyzed for size distribution by an Agilent 2100 Bioanalyzer and quantified using real-time PCR. The whole genomes of the ancestral population and evolved populations were sequenced using an Illumina NovaSeq 6000 instrument.

For data processing, the original data from the Illumina platform were transformed into raw sequenced reads by CASAVA base calling and stored in FASTQ (fq) format, which contained read sequences and the corresponding sequencing quality information of the reads. The base quantity of each sample was 1.7, 1.3, 1.3, 1.2, 1.2, 1.0, 1.0, 1.6, 1.4, 2.0, 1.7, 1.3, and 1.5 billion bp for ancestral, B1-1, B1-2, B1-3, B2-1, B2-2, B2-3, B2-2-1, B2-2-2, B2-2-3, EXP1, EXP2, and EXP3, respectively, and the reads quantity (base quantity divided by the read length) was 11.6, 8.3, 8.9, 7.8, 8.3, 6.8, 7.0, 10.6, 9.6, 13.6, 11.3, 8.9, and 9.8 million reads for ancestral, B1-1, B1-2, B1-3, B2-1, B2-2, B2-3, B2-2-1, B2-2-2, B2-2-3, EXP1, EXP2, and EXP3, respectively. The sequenced data were filtered, and the sequences of adapter and low-quality data were removed, resulting in clean data used for subsequent analysis. Specific processing steps were as follows: we eliminated reads for which low-quality nucleotides (Q-score, ≤5) exceeded a certain threshold (50% of the total base by default); eliminated reads which contained N nucleotides exceeding a certain threshold (10% of the total base by default, where N represents the base cannot be determined); eliminated reads whose overlap with the adapter exceeded a certain threshold (10 bp by default); and finally, filtering the duplications.

### Whole-genome sequencing data analysis.

We performed a genomic comparison between the ancestral and evolved populations to the reference genome. The differences between the populations and the reference genome were obtained by aligning the sample reads with the reference genome (MRSA ATCC 43300 genome downloaded from the ATCC website, January 2022) using BWA mapper V0.7.17 (61). The parameters of BWA were as follows: mem -t 4 -k 32 -M -R. The mapping rate was above 99.85% for all populations. SAMTOOLS V1.9 (62) was used to detect single-nucleotide polymorphisms (SNPs) and small indels (<50 bp) with the following parameters: mpileup -m 2 -F 0.002 -d 10000 -u -L 10000, and call –ploidy 1 -mv -Ov. The detected SNPs were further filtered with QUAL > 30 so that the final SNP list contained high-quality SNPs with high confidence. Subsequently, Integrative Genomics Viewer (IGV) (63) was used to view the aligned sequence and perform further analysis on the identified SNPs/indels (e.g., determination of amino acid substitution, visualizing the effect of base pair deletion to the protein sequence). To verify the results and mine for lower-abundance indels that were not detected by SAMTOOLS, Snippy V4.6.0 (64), a rapid haploid variant calling and core genome alignment software, was used in conjunction to analyze the whole-genome sequencing data. To estimate the proportion of mutants in the population, we used the ratio of the number of alternate reads (reads of the mutation) to the total number of reads at the locus (number of alternate reads + number of reference reads) extracted from Snippy vcf result files. To verify our approach, we isolated three single colonies of a pure *yycH* mutant from the B2-2 population (consisting of ~97.3% *yycH* mutant) by plating on agar and subjecting them to whole-genome sequencing. As expected, the ratio of the number of alternate reads to the total number of reads at the *yycH* gene was 100% for the three *yycH* mutants. The WGS raw data were submitted and are accessible in NCBI BioProject PRJNA798197 (for ancestral, B1-1, B1-2, B1-3, B2-1, B2-2, B2-3, B2-2-1, B2-2-2, and B2-2-3) and PRJNA819400 (for EXP1, EXP3, and EXP3).

### Sample preparation for proteomics.

For proteomics analysis, stationary-phase ancestral, tolerant (B2-2), and resistant (EXP3) populations were treated with daptomycin (30 mg/liter) for 6 h. Similar to our previous work (13), two different strategies were used for the proteomics analysis. First, the proteome sprofile of the tolerant and resistant evolved populations were compared to the ancestral strain as a control to reveal the effect of the mutations on the phenotype of the tolerant and resistant populations. Next, we compared the proteome profile of each population before and after antibiotic treatment to obtain population-specific adaptation strategies toward daptomycin exposure. Three biological replicates were performed for each sample including the control sample.

The cell pellet was suspended in 300 μL of lysis buffer (8 M urea, 50 mM Tris-HCl; pH 8.0), frozen in liquid nitrogen, and sonicated for 10 min. The sample was centrifuged (16,000 × *g* for 10 min) to remove cell debris and insoluble materials. An aliquot of the sample was taken for a bicinchoninic acid protein assay (Pierce). After protein quantification, the sample was reduced with dithiothreitol (0.1 M final concentration) at 37°C for 1 h. For shotgun proteomics, 200 μg of protein was mixed with up to 250 μL of the exchange buffer (6 M urea, 50 mM Tris-HCl [pH 8.0], 600 mM guanidine-HCl), transferred to an Amicon filter device (Millipore, Darmstadt, Germany), and centrifuged (14,000 × *g* for 20 min). The proteins in the filter device were alkylated with iodoacetamide (50 mM in exchange buffer) in the dark for 20 min and then centrifuged (14,000 × *g* for 20 min). To dilute the urea concentration, 250 μL of 50 mM ammonium bicarbonate was added to the filter device and centrifuged (14,000 × *g* for 20 min). This step was repeated once. Proteins were digested by using sequencing-grade modified trypsin (1:50 [wt/wt], Promega, Madison, WI) for 12 h at 37°C. Then, the sample was acidified with 10% formic acid to a final concentration of 0.1% (vol/vol) and centrifuged at 16,000 × *g* for 5 min. Finally, the samples were desalted with a C_18_ reverse-phase ZipTip apparatus (Millipore, Darmstadt, Germany) and dried with a SpeedVac (Eppendorf, Hamburg, Germany) for 30 min.

### Liquid chromatography and mass spectrometry for proteomics.

The samples were reconstituted in 25 μL water-acetonitrile-formic acid in a 97.9:2:0.1 ratio (vol/vol/vol) and processed through a Bruker nanoElute ultra-high-performance liquid chromatograph (UHPLC, Bruker Daltonics, Bremen, Germany) coupled to a hybrid trapped-ion mobility-quadrupole time-of-flight mass spectrometer (TimsTOF Pro, Bruker Daltonics, Bremen, Germany) via a nano-electrospray ion source (Captive Spray, Bruker Daltonics). A volume of 1 μL (approximately 200 ng of the protein digest) was injected into the UHPLC system and separated on an IonOpticks 25 cm Aurora series emitter column with captive spray insert (250 mm by 75 μm internal diameter, 120 Å pore size, 1.6 μm particle size, C_18_) at a flow rate of 0.3 μL/min. The mobile phase composition was 0.1% formic acid in water for solvent A and 0.1% formic acid in acetonitrile for solvent B. The gradient was applied from 2% to 5% solvent B for 0.5 min, from 5% to 30% solvent B for 26.5 min, and then from 30% to 95% solvent B for 0.5 min. In the end, the mobile phase was kept at 95% solvent B for 0.5 min and then decreased to 2% of solvent B for 0.1 min. A 2-min equilibration with 2% solvent B was applied before the next injection.

A detailed description of the Bruker TimsTOF Pro mass spectrometer used in this work can be found in the literature (65, 66). We set the accumulation and ramp time to 100 ms each and recorded mass spectra in the range from *m/z* 100 to 1,700 using the positive electrospray mode. The ion mobility was scanned from 0.85 to 1.30 Vs/cm^2^. The quadrupole isolation width was set to 2 Th for *m/z* <700 and 3 Th for *m/z* >700, and the collision energy was linearly increased from 27 eV to 45 eV as a function of increasing ion mobility. The overall acquisition cycle of 0.53 s comprised one full TIMS-MS scan and four parallel accumulation-serial fragmentation (PASEF) tandem mass spectrometry (MS/MS) scans. Low-abundance precursor ions with an intensity above a threshold of 2,500 counts but below a target value of 20,000 counts were repeatedly scheduled and otherwise dynamically excluded for 0.4 min. The TIMS dimension was calibrated linearly using three selected ions from the Agilent ESI LC/MS tuning mix (*m/z*, 1/K0: [622.0289, 0.9848 Vs cm^−2^], [922.0097, 1.1895 Vs cm^−2^], [1221,9906, 1.3820 Vs cm^−2^]) in positive mode.

### Sequence database searching of proteomics data.

The raw data were converted to mgf files by using Bruker Compass DataAnalysis (version 5.2) and subsequently converted to mzML files by msconvert of the ProteoWizard (version 3.0.20229 64-bit) (67). The mzML files were searched using Comet (version 2021.01 rev.0) (68) with a custom database. Briefly, the genome sequence of S. aureus ATCC 43300 was converted into a protein database using the GeneMark (version 3.25) gene prediction tool (69). The proteins were then annotated using BLASTp (version 2.7.1) from NCBI using S. aureus NCTC 8325 as the protein database. The sequences of common contaminants, such as trypsin and human keratins, and decoy sequences generated by shuffling amino acid sequences between tryptic cleavage sites were added to the database. The decoy sequences in the database are used for the false-discovery rate estimation of the identified peptides. The search parameters criteria were set as follows: 40 ppm peptide mass tolerance, monoisotopic mass type, fully digested enzyme termini, 0.05 amu fragment bin tolerance, 0 amu fragment bin offset, carbamidomethylated cysteine, and oxidated methionine as the fixed and variable modifications, respectively. The search results from Comet were processed by using PeptideProphet (70), iProphet, and ProteinProphet of the Trans-Proteomics Pipeline (TPP) (71) in the decoy-assisted nonparametric mode. Every mzML run was analyzed independently. Protein identifications were filtered at a false discovery rate of 0.01 as predicted by ProteinProphet.

### Label-free quantification of proteomics data by spectral counting.

The proteins identified in at least two out of three biological replicates were used for label-free quantification by spectral counting. The quantification of proteins was given by the normalized spectral abundance factor (NSAF) (72), where the number of peptide-spectrum matches (PSMs) for each protein divided by the length of the corresponding protein was normalized to the total number of PSMs divided by the lengths of protein for all identified proteins. The differentially expressed proteins were filtered by the following cutoff: average spectral counts of at least 3, the *P* values for Student's *t* test on the NSAF values were lower than 0.05, and the fold changes were higher or lower than ±1.5. Moreover, unique proteins that were only detected in the treatment and experimental samples or the control samples were retained for analysis, as they were also likely to have higher or lower expression. To minimize false positives, we further limited our attention to only uniquely detected proteins with spectral counts greater than 3. Here, we assumed that these unique proteins with sufficiently high spectral counts were also induced or upregulated (if detected only in the treatment or experimental samples and not in control samples) or repressed or downregulated (if detected only in control samples and not in treatment samples).

### Bioinformatics analysis.

To highlight potentially important proteins among the differentially expressed proteins, STRING version 11.5 (73) was used to predict the protein-protein interactions and to visualize the interactions. DAVID (Database for Annotation, Visualization and Integrated Discovery) version 6.8 (74) was used for GO and pathway analysis.

### Data availability.

Whole-genome sequence data have been deposited to the BioProject database (NCBI) under the accession numbers PRJNA798197 and PRJNA819400. The mass spectrometry proteomics data have been deposited with ProteomeXchange via the PRIDE repository with the data set identifier PXD031078.
