# Supplementary material for: Mutation in the Two-Component System Regulator YycH Leads to Daptomycin Tolerance in Methicillin-Resistant Staphylococcus aureus upon Evolution with a Population Bottleneck
Source: Microbiol Spectr. 2022 Aug 1;10(4):e01687-22. doi: 10.1128/spectrum.01687-22 (PMC9431245; doi:10.1128/spectrum.01687-22)
Supplement: Supplemental file 1 — Supplemental material. Download spectrum.01687-22-s0001.pdf, PDF file, 1.1 MB [file spectrum.01687-22-s0001.pdf]

Supplementary Information

Mutation in the Two-component System Regulator YycH Led to Daptomycin Tolerance in Methicillin-Resistant *Staphylococcus aureus* upon Evolution with Population Bottleneck

Jordy Evan Sulaiman[†], Long Wu[†], Henry Lam[†, *]

[†] Department of Chemical and Biological Engineering, The Hong Kong University of Science & Technology, Clear Water Bay, Kowloon, Hong Kong

[*] Corresponding author: kehlam@ust.hk

Content:

Supplementary Figures

Fig. S1. Effect of deletions in *yycH, mprF, lacE,* and *yycI* genes on their protein sequence.

Fig S2. Treating the tolerant populations in the exponential phase without population bottlenecks led to the emergence of resistance.

Supplementary Tables

Table S1. MIC of the ancestral and evolved populations towards daptomycin and vancomycin.

Table S2. List of mutations in the evolved populations from whole-genome sequencing.

Table S3. Mutations on specific evolved populations.

Table S4. List of the differentially expressed proteins in the *yycH* tolerant population compared to the ancestral.

Table S5. List of the differentially expressed proteins in the ancestral population upon daptomycin treatment compared to the untreated one.

Table S6. List of the differentially expressed proteins in the *yycH* tolerant population upon daptomycin treatment compared to the untreated one.

Table S7. List of the differentially expressed proteins in the resistant population compared to the ancestral.

Table S8. List of the differentially expressed proteins in the resistant population upon daptomycin treatment compared to the untreated one.

Supplementary Figures

a *yycH:* Two-component system activity regulator *(Early termination)*

Ref: …KVTNI**AIGYEMQDNPDHNHIEVQINSELVPRWYVEYDGEWYVVYNDGRLE**

B2-2: …KVTNI**LSVTKCKIIRIIITLKCRLTVNSYRVGM**

b *mprF:* Phosphatidylglycerol lysyltransferase *(Substitution of 3 AA to 1 AA)*

Ref: …NLT**IVY**DALY…

EXP3: …NLT**N**DALY…

c *lacE:* PTS system lactose specific EIICB component *(Lost 1 AA)*

Ref: …SEIL**DEEE**GRKES…

EXP3: …SEIL**DEE**GRKE…

d *yycI:* WalR/WalK two-component system regulatory protein *(Early termination)*

Ref: …VINARL**GYYSVVNETNVQLLQPNWEIKVKHDGKDKTNTYYVEATNNSPKIINH**

EXP3: …VINARL**VTTQS**

Fig. S1. Effect of deletions in *yycH*, *mprF*, *lacE*, and *yycI* genes on their protein sequence.

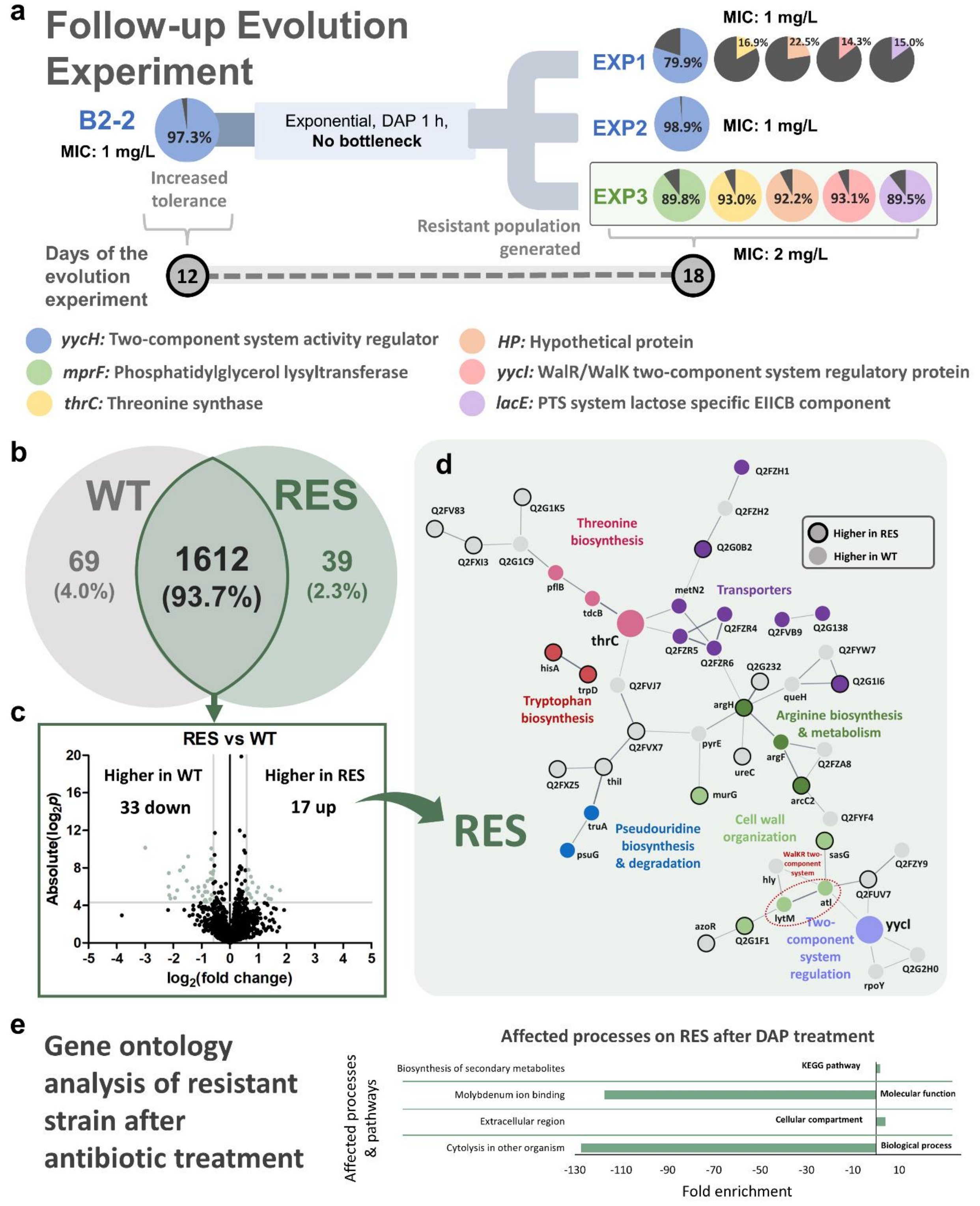

Fig. S2. Treating the tolerant populations in the exponential phase without population bottlenecks led to the emergence of resistance. **a,** Summary of the follow-up evolution experiment. Three parallel B2-2 populations were subjected to evolution experiments by treating them with daptomycin during the exponential phase without population bottlenecks (EXP1, EXP2, and

EXP3). EXP1 gained four additional mutations in the *thrC*, *yycI*, *lacE*, and *hp* genes, EXP2 retained the mutation in *yycH* without additional mutations, and EXP3 lost the *yycH* mutation and gained five new mutations in *mprF*, *yycI*, *thrC*, *lacE*, and *hp* (for details of the mutation, see **Table S2** and **Table S3**). EXP1 and EXP2 retain the tolerance phenotype whereas EXP3 became resistant (2-fold increase in MIC towards daptomycin) due to the well-known resistance mutation in the *mprF* gene. Pie charts show the proportion of specific mutations in the population based on the number of alternate reads divided by the total number of reads at the locus. Black color indicates the % of reference reads, whereas blue, green, red, yellow, purple, and orange colors indicate the % of alternate reads for *yycH*, *mprF*, *yycI*, *thrC*, *lacE*, and *hp* genes. **b,** Venn diagram for proteome comparison of the resistant (EXP3) to the ancestral population. **c,** Volcano plot of the resistant (EXP3) population compared to the ancestral. Differentially expressed proteins (DEPs) are defined to be those with *p*-values below 0.05, and absolute fold change greater than 1.5, corresponding to the colored dots. **d**, Protein-protein interaction network of the DEPs of the tolerant population compared to the ancestral population, as predicted by STRING v11.5. The lines represent protein interaction (thicker lines mean higher confidence), and the dots in different colors represent different protein functions. Nodes without function enrichment are colored gray. Nodes with black outlines are proteins with higher expression in the resistant population, and nodes with no outlines are proteins with higher expression in the ancestral population. Node corresponding to the mutated genes in the resistant population (*yycI* and *thrC*) are shown with twice the size of the other nodes. Nodes that belong to the WalKR two-component system are marked. **e**, Gene Ontology (GO) analysis, and pathway enrichment study (KEGG) by DAVID of the DEPs of the resistant population after daptomycin treatment compared to those before treatment. Fold enrichment is defined as the ratio of the proportion of the input information to the background information. Negative values mean down-regulated and positive values mean up-regulated.

Supplementary Tables

Table S1. MIC of the ancestral and evolved populations towards daptomycin and vancomycin.

Strain	MIC (mg/L)	
	Daptomycin	Vancomycin
Ancestral	1.0	1.5
B1-1	1.0	4.0
B1-2	1.0	4.0
B1-3	1.0	4.0
B2-1	1.0	4.0
B2-2	1.0	4.0
B2-3	1.0	4.0
B2-2-1	1.0	4.0
B2-2-2	1.0	4.0
B2-2-3	1.0	4.0
EXP1	1.0	4.0
EXP2	1.0	4.0
EXP3	2.0	4.0

Table S2. List of mutations in the evolved populations from whole-genome sequencing.

Genomic position	Mutation	Amino acid substitution	Gene	Annotation
690,846	TATACAA > T	Substitution of 3 AA to 1 AA	*mprF*	Phosphatidylglycerol lysyltransferase
728,271	C > T	Cys36Tyr	*thrC*	Threonine synthase
732,208	G > A	Val5Ile	*hp*	Hypothetical protein
2,091,253	CG > C	Early termination	*yycI*	WalR/WalK two-component system regulatory protein
2,092,032	GC > G	Early termination	*yycH*	Two component system activity regulator YycH
2,660,853	TGAA > T	Deletion of 1 AA	*lacE*	PTS system lactose specific EIICB component

Table S3. Mutations on specific evolved populations.

Mutated gene	B1-1	B1-2	B1-3	B2-1	B2-2	B2-3	B2-2-1	B2-2-2	B2-2-3	EXP1	EXP2	EXP3
mprF	−	−	−	−	−	−	−	−	−	−	−	+
thrC	+	+	+	−	−	+	−	−	−	+	−	+
hp	+	+	+	−	−	+	−	−	−	+	−	+
yycI	+	+	+	−	−	+	−	−	−	+	−	+
yycH	+	+	+	+	+	+	+	+	+	+	+	−
lacE	+	+	+	−	−	+	−	−	−	+	−	+
**Phenotype**	TOL	TOL	TOL	TOL	TOL	TOL	TOL	TOL	TOL	TOL	TOL	RES

Table S4. List of the differentially expressed proteins in the *yycH* tolerant population compared to the ancestral.

Protein	*p-value*	Fold change / (average PSM)	Gene	Protein
Q2G1K5	-	(12)	*SAOUHSC_00117*	Capsular polysaccharide biosynthesis protein Cap5D, putative
Q2FYR7	-	(3.33)	*trpD*	Anthranilate phosphoribosyltransferase
Q2FV27	0.025062	3.60	*SAOUHSC_02912*	3-dmu-9_3-mt domain-containing protein
Q2G1K4	0.016403	3.58	*SAOUHSC_00118*	UDP-galactose 4-epimerase
Q2FUY6	0.008056	3.43	*SAOUHSC_02958*	Alkaline phosphatase III, putative
Q2G2B2	0.012768	3.14	*sasG*	Surface protein G
Q2G1I7	0.036956	3.13	*SAOUHSC_00135*	Uncharacterized protein
Q2FZS2	0.023987	2.43	*SAOUHSC_00918*	Truncated MHC class II analog protein
Q2FXW2	0.013621	2.36	*SAOUHSC_01719*	UPF0473 protein SAOUHSC_01719
Q2FYY9	0.04896	2.35	*hflX*	GTPase HflX (GTP-binding protein HflX)
Q2FYQ0	0.032427	2.24	*pstB*	Phosphate import ATP-binding protein PstB (EC 7.3.2.1)
Q2G0J7	0.02738	2.19	*ung*	Uracil-DNA glycosylase (UDG) (EC 3.2.2.27)
Q2G0T9	0.033551	2.15	*SAOUHSC_00438*	Alpha amylase family protein, putative
Q2FYE5	0.036598	2.08	*SAOUHSC_01508*	Uncharacterized protein
Q2G174	0.033338	2.04	*SAOUHSC_00279*	DUF4467 domain-containing protein
gene_2701	0.047163	2.01	gene_2701	Uncharacterized protein
gene_2701	0.037875	1.98	*SAOUHSC_01888*	Riboflavin synthase, alpha subunit (EC 2.5.1.9)
Q2FWX4	0.001354	1.95	*SAOUHSC_02147*	DUF4097 domain-containing protein
Q2G1K7	0.020636	1.78	*SAOUHSC_00115*	Non-specific protein-tyrosine kinase (EC 2.7.10.2)
Q2G1V4	0.003332	1.76	*SAOUHSC_00333*	ABC transporter, ATP-binding protein, putative
Q2FXK4	0.004063	1.75	*SAOUHSC_01830*	GP-PDE domain-containing protein
Q2FVZ7	0.016087	1.74	*SAOUHSC_02523*	Uncharacterized protein

Q2FW49	0.035256	1.69	*SAOUHSC_02468*	Acetolactate synthase, putative (EC 2.2.1.6)
Q2FZU7	0.023373	1.69	*SAOUHSC_00893*	FMN oxidoreductase, putative
gene_2653	0.032227	1.69	gene_2653	Uncharacterized protein
Q2FWG3	0.021003	1.68	*thiE*	Thiamine-phosphate synthase (TP synthase) (TPS) (EC 2.5.1.3)
Q2G0F2	0.009609	1.64	*SAOUHSC_00617*	Uncharacterized protein
Q2FV99	0.044784	1.63	*srtA*	Sortase A (EC 3.4.22.70) (Surface protein sorting A)
Q2FWE7	0.021434	1.56	*atpH*	ATP synthase subunit delta (ATP synthase F(1) sector subunit delta)
Q2FVM1	0.030985	1.54	*SAOUHSC_02681*	Nitrate reductase (quinone) (EC 1.7.5.1)
Q2G2P7	0.03892	1.51	*hutH*	Histidine ammonia-lyase (Histidase) (EC 4.3.1.3)
Q2G161	0.024698	0.66	*SAOUHSC_00294*	Uncharacterized protein
Q2FW21	0.020325	0.66	*rplF*	50S ribosomal protein L6
Q2G0X1	0.011858	0.66	*SAOUHSC_00402*	Uncharacterized lipoprotein SAOUHSC_00402
Q2FW86	0.032368	0.64	*SAOUHSC_02417*	Iron-sulfur cluster carrier protein
Q2FY14	0.048241	0.63	*SAOUHSC_01660*	GTP cyclohydrolase 1 type 2 homolog
Q2G031	0.02189	0.63	*pgk*	Phosphoglycerate kinase (EC 2.7.2.3)
Q2G1C5	0.024923	0.60	*SAOUHSC_00200*	Membrane protein, putative
Q2FXF9	0.011801	0.60	*SAOUHSC_01889*	Riboflavin biosynthesis protein RibD (EC 3.5.4.26)
Q2G232	0.046408	0.59	*SAOUHSC_00450*	Orn/Lys/Arg decarboxylase, putative
Q2FWW1	0.026812	0.58	*SAOUHSC_02161*	MHC class II analog protein
Q2G298	0.001118	0.58	*rsfS*	Ribosomal silencing factor RsfS
Q2G0I6	0.033197	0.58	*SAOUHSC_00579*	Phosphomevalonate kinase (EC 2.7.4.2)
Q2FVK1	0.047863	0.57	*hlgB*	Gamma-hemolysin component B (H-gamma-1) (H-gamma-I) (Leukocidin f subunit)
Q2FV44	0.039113	0.57	*SAOUHSC_02895*	NmrA domain-containing protein
Q2G1N4	0.017014	0.56	*SAOUHSC_00074*	Periplasmic binding protein, putative
Q2FVT8	0.019189	0.55	*SAOUHSC_02604*	Uncharacterized protein
Q2FZK7	0.006945	0.54	*atl*	Bifunctional autolysin [Includes: N-acetylmuramoyl-L-alanine amidase (EC 3.5.1.28)
Q2FVJ7	0.005563	0.53	*bioB*	Biotin synthase (EC 2.8.1.6)
Q2G2J2	0.000337	0.52	*ssaA2*	Staphylococcal secretory antigen ssaA2
gene_1727	7.77E-05	0.47	gene_1727	Uncharacterized protein
gene_1918	0.043887	0.47	gene_1918	Uncharacterized protein
Q2FWP0	0.006928	0.47	*SAOUHSC_02241*	Uncharacterized leukocidin-like protein 1
Q2FV55	0.015232	0.47	*ssaA*	Staphylococcal secretory antigen SsaA
Q2FXP0	0.003304	0.46	*SAOUHSC_01796*	DNA-(apurinic or apyrimidinic site) lyase MutM (EC 3.2.2.23) (EC 4.2.99.18)
Q2G0W1	0.039252	0.45	*dabA*	Probable inorganic carbon transporter subunit DabA
Q2FVK5	0.017503	0.44	*sbi*	Immunoglobulin-binding protein Sbi
Q2G1U2	0.048877	0.43	*SAOUHSC_01174*	3-dmu-9_3-mt domain-containing protein

Q2FWN9	0.016041	0.39	*SAOUHSC_02243*	Uncharacterized leukocidin-like protein 2
Q2FXG1	0.04176	0.39	*ribBA*	Riboflavin biosynthesis protein RibBA (EC 4.1.99.12)
Q2G065	0.004508	0.39	*SAOUHSC_00756*	Uncharacterized protein
Q2FZX3	0.046518	0.36	*SAOUHSC_00862*	Uncharacterized protein
Q2FWI8	0.026445	0.33	*mazF*	Endoribonuclease MazF (EC 3.1.-.-) (Toxin MazF) (mRNA interferase MazF)
Q2FX00	0.015829	0.32	*gatD*	Lipid II isoglutaminyl synthase (glutamine-hydrolyzing) subunit GatD (EC 6.3.5.13)
Q2FV28	0.026843	0.30	*queH*	Epoxyqueuosine reductase QueH (EC 1.17.99.6) (Queuosine biosynthesis protein QueH)
Q2G1D3	0.031992	0.27	*SAOUHSC_00192*	Coagulase
Q2G0U9	0.04102	0.24	*sle1*	N-acetylmuramoyl-L-alanine amidase sle1 (EC 3.5.1.28)
Q2FYG6	0.045823	0.19	*SAOUHSC_01486*	Heptaprenyl diphosphate syntase component II, putative (EC 2.5.1.30)
Q2FVQ4	0.049339	0.18	*SAOUHSC_02648*	L-lactate permease
Q2FV26	-	(-3)	*SAOUHSC_02913*	HTH tetR-type domain-containing protein
Q2FWJ9	-	(-3)	*ilvA*	L-threonine dehydratase biosynthetic IlvA (EC 4.3.1.19) (Threonine deaminase)
Q2FWV6	-	(-3)	*scn*	Staphylococcal complement inhibitor (SCIN)
P69848	-	(-3)	*moaA*	GTP 3',8-cyclase (EC 4.1.99.22) (Molybdenum cofactor biosynthesis protein A)
Q2FZQ1	-	(-3)	*SAOUHSC_00949*	Uncharacterized protein
Q2FY86	-	(-3.33)	*SAOUHSC_01578*	Conserved hypothetical phage protein
Q2G1V8	-	(-3.33)	*SAOUHSC_02575*	Uncharacterized protein
Q2FWN1	-	(-3.33)	*SAOUHSC_02257*	Uncharacterized protein
Q2FYW7	-	(-3.33)	*SAOUHSC_01306*	Uncharacterized protein
Q2G0V7	-	(-3.33)	*SAOUHSC_00417*	Hydrolase_4 domain-containing protein
Q2G1E4	-	(-3.66)	*SAOUHSC_00181*	AP_endonuc_2 domain-containing protein
gene_1967	-	(-3.66)	*gene_1967*	Uncharacterized protein
Q2FXU8	-	(-3.66)	*SAOUHSC_01734*	AAA domain-containing protein
Q2FY23	-	(-3.66)	*SAOUHSC_01650*	5-formyltetrahydrofolate cyclo-ligase (EC 6.3.3.2)
Q2G1L5	-	(-4)	*SAOUHSC_00107*	Uncharacterized protein
Q2G0Y5	-	(-4)	*SAOUHSC_00376*	Uncharacterized protein
Q2G0E8	-	(-4.33)	*SAOUHSC_00658*	Phosphoenolpyruvate--glycerone phosphotransferase (EC 2.7.1.121)
Q9ZNI1	-	(-4.33)	*lytN*	Probable cell wall hydrolase LytN (EC 3.-.-.-)
Q2FWN2	-	(-4.33)	*SAOUHSC_02256*	Abortive infection protein
Q2G1A8	-	(-4.66)	*SAOUHSC_00237*	Methyltransf_25 domain-containing protein
Q2FV69	-	(-5)	*SAOUHSC_02867*	HTH tetR-type domain-containing protein
Q2G012	-	(-5.33)	*emp*	Extracellular matrix protein-binding protein emp
gene_2767	-	(-6)	*gene_2767*	Uncharacterized protein

Q2G2X5	-	(-6)	*SAOUHSC_00642*	Transport permease protein
Q2G1X0	-	(-6.33)	*hly*	Alpha-hemolysin (Alpha-HL) (Alpha-toxin)
Q2FVP9	-	(-6.66)	*SAOUHSC_02653*	GCN5-related N-acetyltransferase
Q2FY44	-	(-7)	*SAOUHSC_01622*	Alkaline shock protein 23
Q2FVB9	-	(-8.33)	*SAOUHSC_02815*	MFS domain-containing protein
Q2FY74	-	(-8.33)	*xerD*	Tyrosine recombinase XerD
Q2G2U3	-	(-17.33)	*yycH*	YycH domain-containing protein

Table S5. List of the differentially expressed proteins in the ancestral population upon daptomycin treatment compared to the untreated one.

Uniprot ID	*p-value*	Fold change / (average PSM)	Gene	Protein
Q2FVU5	-	(21.66)	*SAOUHSC_02596*	Uncharacterized protein
Q2G0V3	-	(21)	*SAOUHSC_00422*	Trans-sulfuration enzyme family protein, putative
Q2G1F1	-	(13.66)	*SAOUHSC_00174*	Lysostaphin (EC 3.4.24.75)
Q2FUS7	-	(12.66)	*trhO*	tRNA uridine(34) hydroxylase (EC 1.14.-.-) (tRNA hydroxylation protein O)
Q2FWU3	-	(11.66)	*SAOUHSC_02182*	Lysostaphin (EC 3.4.24.75)
Q2FUS8	-	(10.33)	*drp35*	Lactonase drp35 (EC 3.1.1.-)
Q2G2N5	-	(10)	*SAOUHSC_01354*	Sodium:alanine symporter family protein, putative
Q2FWZ4	-	(9)	*SAOUHSC_02112*	Uncharacterized protein
Q2FZU2	-	(9)	*argH*	Argininosuccinate lyase (ASAL) (EC 4.3.2.1) (Arginosuccinase)
Q2FXQ9	-	(8.66)	*hemA*	Glutamyl-tRNA reductase (GluTR) (EC 1.2.1.70)
Q2G0D5	-	(8.33)	*SAOUHSC_00670*	Uncharacterized protein
Q2FYM9	-	(8)	*acyP*	Acylphosphatase (EC 3.6.1.7) (Acylphosphate phosphohydrolase)
Q2FVS9	-	(7.66)	*SAOUHSC_02613*	MOSC domain-containing protein
Q2FZ56	-	(7)	*fapR*	Transcription factor FapR (Fatty acid and phospholipid biosynthesis regulator)
Q2FVN8	-	(6.66)	*SAOUHSC_02664*	Transcriptional regulator, putative
Q2FWU4	-	(6.66)	*SAOUHSC_02181*	Phi PVL orfs 18-19-like protein
Q2FXI2	-	(6.66)	*trmB*	tRNA (guanine-N(7)-)-methyltransferase (EC 2.1.1.33)
Q2G0B8	-	(6.33)	*SAOUHSC_00687*	N-acetyltransferase domain-containing protein
Q2G148	-	(6.33)	*SAOUHSC_00307*	Deacetylase sirtuin-type domain-containing protein
Q2G2K5	-	(6.33)	*ureC*	Urease subunit alpha (EC 3.5.1.5) (Urea amidohydrolase subunit alpha)
Q2G0U3	-	(6)	*SAOUHSC_00434*	Transcriptional regulator, lysR family, putative
gene_673	-	(6)	*gene_673*	Uncharacterized protein

Q2G0E6	-	(5.66)	*SAOUHSC_00660*	Uncharacterized protein
Q2G2S1	-	(5.66)	*SAOUHSC_01536*	ATP-dependent Clp protease proteolytic subunit
Q2FWX0	-	(5.66)	*SAOUHSC_02151*	Uncharacterized protein
Q2FZP3	-	(5.66)	*SAOUHSC_00957*	Uncharacterized protein
Q2G090	-	(5.33)	*SAOUHSC_00730*	DNA helicase (EC 3.6.4.12)
Q2G240	-	(5.33)	*SAOUHSC_00706*	Lactose phosphotransferase system repressor
Q2FXW9	-	(5.33)	*SAOUHSC_01711*	AHS2 domain-containing protein
Q2G0J9	-	(5)	*SAOUHSC_00561*	Protein VraX
Q2G185	-	(5)	*essB*	Type VII secretion system protein EssB
gene_1926	-	(5)	*gene_1926*	Uncharacterized protein
Q2FY76	-	(4.66)	*scpA*	Segregation and condensation protein A
Q2FYE9	-	(4.66)	*SAOUHSC_01503*	HTH_40 domain-containing protein
Q2FVH2	-	(4.66)	*SAOUHSC_02740*	Drug transporter, putative
Q2FYN7	-	(4.33)	*dapH*	2,3,4,5-tetrahydropyridine-2,6-dicarboxylate N-acetyltransferase (EC 2.3.1.89)
Q2G1K5	-	(4.33)	*SAOUHSC_00117*	Capsular polysaccharide biosynthesis protein Cap5D, putative
Q2FVQ9	-	(4.33)	*hssR*	Heme response regulator HssR
Q2G1F8	-	(4.33)	*SAOUHSC_00167*	Peptide ABC transporter, ATP-binding protein, putative
Q2FZ62	-	(4.33)	*rpe*	Ribulose-phosphate 3-epimerase (EC 5.1.3.1)
Q2FYL1	-	(4.33)	*SAOUHSC_01429*	UPF0346 protein SAOUHSC_01429
Q2G0T6	-	(4)	*SAOUHSC_00441*	N-acetyltransferase domain-containing protein
Q2FWT7	-	(4)	*SAOUHSC_02188*	Phage head-tail adaptor, putative
Q2G2E0	-	(3.66)	*SAOUHSC_00633*	Na_H_Exchanger domain-containing protein
Q2G0K3	-	(3.66)	*SAOUHSC_00557*	Acyl-CoA synthetase (Putative long chain fatty acid-CoA ligase VraA)
Q2FV45	-	(3.66)	*SAOUHSC_02894*	VOC domain-containing protein
Q2FX06	-	(3.66)	*SAOUHSC_02101*	Uncharacterized protein
Q2FUV7	-	(3.33)	*SAOUHSC_02994*	Uncharacterized protein
Q2FYR7	-	(3.33)	*trpD*	Anthranilate phosphoribosyltransferase (EC 2.4.2.18)
Q2G1I3	-	(3.33)	*SAOUHSC_00139*	Uncharacterized protein
Q2G0I9	-	(3.33)	*lipL*	Octanoyl-[GcvH]:protein N-octanoyltransferase (EC 2.3.1.204)
Q2FVF8	-	(3.33)	*SAOUHSC_02756*	Putative mRNA interferase YoeB (Toxin YoeB)
Q2G1E3	-	(3.33)	*SAOUHSC_00182*	Uncharacterized protein
Q2FZR7	-	(3.33)	*SAOUHSC_00923*	ABC transmembrane type-1 domain-containing protein
Q2FVW6	-	(3.33)	*SAOUHSC_02557*	Urea transporter, putative
Q2G0F4	-	(3.33)	*SAOUHSC_00615*	Haloacid dehalogenase-like hydrolase, putative
Q2FW59	-	(3)	*SAOUHSC_02458*	DUF3885 domain-containing protein
Q2FVE1	-	(3)	*SAOUHSC_02773*	Transporter, putative
Q2FWR9	-	(3)	*SAOUHSC_02211*	Phi PVL orf 50-like protein

gene_1917	-	(3)	*gene_1917*	Uncharacterized protein
Q2FYW6	-	(3)	*SAOUHSC_01307*	Beta_elim_lyase domain-containing protein
Q2FV94	-	(3)	*SAOUHSC_02840*	L-serine deaminase
Q2FYV2	-	(3)	*thrB*	Homoserine kinase (HK) (HSK) (EC 2.7.1.39)
Q2FXZ0	0.001273	12.34	*hrcA*	Heat-inducible transcription repressor HrcA
Q2FXR9	0.004925	9.60	*SAOUHSC_01766*	Folylpolyglutamate synthase/dihydrofolate synthase, putative (EC 6.3.2.17)
Q2FZ44	0.003059	7.25	*rimM*	Ribosome maturation factor RimM
Q2G275	0.029246	6.31	*recF*	DNA replication and repair protein RecF
Q2G238	0.038681	5.36	*SAOUHSC_00707*	Tagatose-6-phosphate kinase (EC 2.7.1.144)
Q2FVQ8	0.009336	5.02	*hssS*	Heme sensor protein HssS (EC 2.7.13.3)
Q2FVY3	0.006039	4.95	*SAOUHSC_02538*	Molybdopterin synthase sulfur carrier subunit
Q2FVL4	0.007018	4.92	*SAOUHSC_02697*	Amino acid ABC transporter, ATP-binding protein, putative
Q2FW04	0.000627	4.80	*SAOUHSC_02516*	Uncharacterized protein
Q2FWW7	0.012607	4.46	*SAOUHSC_02154*	ABC transporter, ATP-binding protein, putative
Q2G147	0.015011	4.19	*SAOUHSC_00308*	Lipoate--protein ligase (EC 6.3.1.20)
Q2G2L4	0.003752	4.12	*SAOUHSC_02814*	Uncharacterized protein
Q2FXV7	0.01338	4.05	*SAOUHSC_01724*	Uncharacterized protein
Q2FW06	0.000949	4.01	*rplC*	50S ribosomal protein L3
Q2FYH0	0.011434	3.86	*aroB*	3-dehydroquinate synthase (DHQS) (EC 4.2.3.4)
Q2FZ30	0.004175	3.81	*xerC*	Tyrosine recombinase XerC
Q2G0J7	0.034547	3.72	*ung*	Uracil-DNA glycosylase (UDG) (EC 3.2.2.27)
Q2FV83	0.011818	3.60	*SAOUHSC_02852*	HTH lysR-type domain-containing protein
Q2FX99	0.04545	3.57	*SAOUHSC_01978*	UPF0754 membrane protein SAOUHSC_01978
Q2FUY1	0.03333	3.54	*arcR*	HTH-type transcriptional regulator ArcR
Q2FVA6	0.027827	3.52	*SAOUHSC_02827*	Uncharacterized protein
Q2G098	0.024586	3.48	*SAOUHSC_00723*	Chorismate_bind domain-containing protein
Q2FYQ0	0.003196	3.46	*pstB*	Phosphate import ATP-binding protein PstB (EC 7.3.2.1)
Q2FXL4	0.00049	3.45	*SAOUHSC_01821*	N6_Mtase domain-containing protein
Q2FVS3	0.000185	3.43	*SAOUHSC_02619*	ABC transporter domain-containing protein
Q2FXU8	0.017445	3.42	*SAOUHSC_01734*	AAA domain-containing protein
Q2FW01	0.014133	3.42	*SAOUHSC_02519*	N-acetyltransferase domain-containing protein
Q2FY48	0.016301	3.40	*SAOUHSC_01618*	Geranyltranstransferase, putative (EC 2.5.1.10)
Q2G1I7	0.013503	3.24	*SAOUHSC_00135*	Uncharacterized protein
Q2G0D8	0.004203	3.21	*SAOUHSC_00667*	Putative hemin import ATP-binding protein HrtA
Q9F1K0	0.002896	3.16	*dnaE*	DNA polymerase III subunit alpha (EC 2.7.7.7)
Q2G0T9	0.031472	3.16	*SAOUHSC_00438*	Alpha amylase family protein, putative
Q2FXY9	0.000269	3.13	*SAOUHSC_01686*	Heme chaperone HemW

Q2FXM0	0.001385	3.10	*SAOUHSC_01815*	UPF0173 metal-dependent hydrolase SAOUHSC_01815
Q2FW52	0.006214	3.08	*SAOUHSC_02465*	Uncharacterized hydrolase SAOUHSC_02465 (EC 3.-.-.-)
Q2FZP7	0.007658	3.06	*ugtP*	Processive diacylglycerol beta-glucosyltransferase (EC 2.4.1.315)
Q2FV59	0.011983	3.04	*crtM*	4,4'-diapophytoene synthase (DAP synthase) (EC 2.5.1.96) (C30 carotenoid synthase) (Dehydrosqualene synthase)
Q2G0B3	0.005639	3.04	*SAOUHSC_00692*	Uncharacterized protein
Q2G1G5	0.033321	3.00	*SAOUHSC_00158*	PTS system MurNAc-GlcNAc-specific EIIBC component
Q2FV29	0.002049	2.96	*SAOUHSC_02910*	Uncharacterized protein
Q2FXW4	0.020049	2.89	*SAOUHSC_01717*	Uncharacterized protein
Q2G0V7	0.011534	2.87	*SAOUHSC_00417*	Hydrolase_4 domain-containing protein
Q2FWL0	0.001631	2.86	*SAOUHSC_02279*	TsaD domain-containing protein
Q2FXT9	0.001785	2.79	*SAOUHSC_01744*	Single-stranded-DNA-specific exonuclease RecJ
Q2FXL3	0.001847	2.71	*tpx*	Thiol peroxidase (Tpx) (EC 1.11.1.24) (Peroxiredoxin tpx) (Prx) (Thioredoxin peroxidase)
Q2G0L7	0.026458	2.69	*azo1*	FMN-dependent NADPH-azoreductase (EC 1.7.-.-)
Q2G1Z3	0.001577	2.68	*SAOUHSC_00652*	Iron compound ABC transporter, ATP-binding protein
Q2FUS9	0.002567	2.65	*SAOUHSC_03022*	UPF0312 protein SAOUHSC_03022
Q2FX07	0.001959	2.64	*SAOUHSC_02100*	DUF2154 domain-containing protein
Q2FVR4	0.016429	2.60	*SAOUHSC_02638*	DUF218 domain-containing protein
P0A0K3	0.008115	2.59	*SAOUHSC_02013*	Uncharacterized protein SAOUHSC_02013 (ORF1)
Q9EZ12	0.01412	2.59	*dapA*	4-hydroxy-tetrahydrodipicolinate synthase (HTPA synthase) (EC 4.3.3.7)
Q2G2V3	0.002075	2.58	*menE*	2-succinylbenzoate--CoA ligase (EC 6.2.1.26) (o-succinylbenzoyl-CoA synthetase) (OSB-CoA synthetase)
Q2FZ12	0.000155	2.54	*SAOUHSC_01259*	HTH cro/C1-type domain-containing protein
Q93Q23	0.001039	2.52	*mgt*	Monofunctional glycosyltransferase (MGT) (EC 2.4.1.129) (Peptidoglycan TGase)
Q2FYY9	0.026086	2.51	*hflX*	GTPase HflX (GTP-binding protein HflX)
Q2FVX7	0.012073	2.44	*SAOUHSC_02545*	ThiF domain-containing protein
Q2FXN2	0.01687	2.44	*SAOUHSC_01803*	AA_permease domain-containing protein
Q2G1F0	0.00725	2.43	*SAOUHSC_00175*	Multiple sugar-binding transport ATP-binding protein, putative
Q2FZL6	0.010356	2.43	*menH*	Putative 2-succinyl-6-hydroxy-2,4-cyclohexadiene-1-carboxylate synthase (SHCHC synthase) (EC 4.2.99.20)
Q2G0E8	0.004656	2.43	*SAOUHSC_00658*	Phosphoenolpyruvate--glycerone phosphotransferase (EC 2.7.1.121)
Q2FZ05	0.003357	2.41	*SAOUHSC_01266*	Uncharacterized protein
Q2G0L3	0.015441	2.39	*SAOUHSC_00547*	Glycos_transf_1 domain-containing protein

Q2FWF1	8.8E-05	2.32	*atpC*	ATP synthase epsilon chain (ATP synthase F1 sector epsilon subunit) (F-ATPase epsilon subunit)
Q2G263	0.023264	2.29	*SAOUHSC_00096*	Transcriptional regulator, GntR family, putative
Q2G0A6	0.015163	2.25	*SAOUHSC_00699*	Deoxyribodipyrimidine photolyase, putative (EC 4.1.99.3)
Q2FZP4	3.51E-05	2.24	*prfC*	Peptide chain release factor 3 (RF-3)
Q2FYT0	0.011467	2.23	*SAOUHSC_01346*	Glycine betaine transporter, putative
Q2G1J6	0.014772	2.21	*SAOUHSC_00126*	Capsular polysaccharide biosynthesis protein Cap8M
Q2G1E9	0.019061	2.21	*SAOUHSC_00176*	Maltodextrin-binding protein
Q2FY33	0.000328	2.20	*gcvT*	Aminomethyltransferase (EC 2.1.2.10) (Glycine cleavage system T protein)
Q2FZZ2	0.044264	2.19	*metN2*	Methionine import ATP-binding protein MetN 2 (EC 7.4.2.11)
Q2FY44	0.010594	2.18	*SAOUHSC_01622*	Alkaline shock protein 23
Q2FVD4	0.001022	2.16	*SAOUHSC_02779*	Uncharacterized protein
gene_2450	0.00176	2.14	gene_2450	Uncharacterized protein
Q2G2H8	0.000551	2.14	*mnhE1*	Na(+)/H(+) antiporter subunit E1 (Mnh complex subunit E1)
Q2FX94	0.000462	2.13	*fumC*	Fumarate hydratase class II (Fumarase C) (EC 4.2.1.2) (Aerobic fumarase) (Iron-independent fumarase)
Q2FYU2	0.013644	2.12	*SAOUHSC_01332*	Uncharacterized protein
Q2FY71	0.007417	2.08	*SAOUHSC_01594*	Aldo_ket_red domain-containing protein
Q2FXH7	0.02256	2.08	*SAOUHSC_01870*	Pseudouridine synthase (EC 5.4.99.-)
Q2FWY3	0.027184	2.07	*SAOUHSC_02138*	Pectate_lyase_3 domain-containing protein
Q2G1U2	0.018054	2.06	*SAOUHSC_01174*	3-dmu-9_3-mt domain-containing protein
Q2FVX9	0.029558	2.01	*moaC*	Cyclic pyranopterin monophosphate synthase (EC 4.6.1.17) (Molybdenum cofactor biosynthesis protein C)
Q2G0G5	0.004764	2.01	*SAOUHSC_00604*	FMN-dependent NADPH-azoreductase (NADPH-dependent flavo-azoreductase) (NADPH-flavin azoreductase)
Q2G1D0	0.001349	1.99	*SAOUHSC_00195*	Acetoacetyl-CoA thiolase (EC 2.3.1.9) (Probable acetyl-CoA acyltransferase)
Q2G095	0.049787	1.98	*SAOUHSC_00726*	AHS1 domain-containing protein
Q2FYY8	0.003313	1.94	*SAOUHSC_01284*	Uncharacterized protein
Q2FZ73	0.006508	1.94	*carA*	Carbamoyl-phosphate synthase small chain (EC 6.3.5.5)
Q2FUW7	0.022598	1.94	*gtfA*	UDP-N-acetylglucosamine--peptide N-acetylglucosaminyltransferase GtfA subunit (EC 2.4.1.-)
Q2FYL5	0.002193	1.94	*murG*	UDP-N-acetylglucosamine--N-acetylmuramyl-(pentapeptide) pyrophosphoryl-undecaprenol N-acetylglucosamine transferase
Q2FX95	0.007722	1.92	*SAOUHSC_01982*	Ribosomal large subunit pseudouridine synthase, RluD subfamily, putative (EC 4.2.1.70)
Q2FZL8	0.041412	1.91	*SAOUHSC_00982*	Isochorismate synthase (EC 5.4.4.2)

Q2G0N7	0.003368	1.90	*SAOUHSC_00523*	MTS domain-containing protein
Q2G157	0.00081	1.89	*nanE*	Putative N-acetylmannosamine-6-phosphate 2-epimerase (EC 5.1.3.9)
Q2FVP8	0.012191	1.89	*SAOUHSC_02654*	Ferredoxin--NADP reductase (FNR) (Fd-NADP(+) reductase) (EC 1.18.1.2)
Q2G2L6	0.043914	1.88	*SAOUHSC_02812*	Uncharacterized protein
Q2FZN7	0.000289	1.87	*SAOUHSC_00963*	Lipoate--protein ligase (EC 6.3.1.20)
Q2G0Q0	0.015011	1.87	*pdxT*	Pyridoxal 5'-phosphate synthase subunit PdxT (EC 4.3.3.6)
Q2FZ78	0.000466	1.85	*SAOUHSC_01163*	Pseudouridine synthase (EC 5.4.99.-)
Q2FZX2	0.032563	1.85	*SAOUHSC_00863*	Uncharacterized protein
Q2FZY5	0.044456	1.84	*SAOUHSC_00849*	Probable cysteine desulfurase (EC 2.8.1.7)
Q2FV44	0.009726	1.83	*SAOUHSC_02895*	NmrA domain-containing protein
Q2FZA9	0.016435	1.83	*arcC1*	Carbamate kinase 1 (EC 2.7.2.2)
Q2G0D9	0.010131	1.82	*graS*	Sensor histidine kinase GraS (EC 2.7.13.3) (Glycopeptide resistance-associated protein S)
Q2G0E7	0.028955	1.82	*SAOUHSC_00659*	Uncharacterized protein
gene_2710	0.032292	1.81	gene_2710	Uncharacterized protein
Q2G1V4	0.002484	1.80	*SAOUHSC_00333*	ABC transporter, ATP-binding protein, putative
Q2FXU7	0.007552	1.80	*SAOUHSC_01735*	ThiF domain-containing protein
gene_2767	0.034213	1.80	gene_2767	Uncharacterized protein
Q2FY37	0.001298	1.80	*lipM*	Octanoyltransferase LipM (EC 2.3.1.181) (Octanoyl-[acyl-carrier-protein]:[GcvH] N-octanoyltransferase)
Q2FVV8	0.015544	1.79	*SAOUHSC_02583*	Transcriptional regulator, putative
Q2FVX2	0.037665	1.79	*SAOUHSC_02551*	N-acetyltransferase domain-containing protein
Q2FXG3	0.004975	1.77	*SAOUHSC_01884*	Proline dehydrogenase (EC 1.5.5.2)
Q2FZD3	0.013032	1.77	*mutS2*	Endonuclease MutS2 (EC 3.1.-.-)
Q2FZU9	0.002827	1.77	*SAOUHSC_00891*	Putative peptidyl-prolyl cis-trans isomerase (PPIase) (EC 5.2.1.8) (Rotamase)
Q2FV60	0.006992	1.75	*crtN*	4,4'-diapophytoene desaturase (4,4'-diaponeurosporene-forming) (EC 1.3.8.-) (Dehydrosqualene desaturase)
Q2FYN6	0.014627	1.74	*SAOUHSC_01399*	Uncharacterized hydrolase SAOUHSC_01399 (EC 3.-.-.-)
Q2FZD8	0.018626	1.73	*pheT*	Phenylalanine--tRNA ligase beta subunit (EC 6.1.1.20) (Phenylalanyl-tRNA synthetase beta subunit) (PheRS)
Q2FW57	0.004566	1.72	*SAOUHSC_02460*	Aldo_ket_red domain-containing protein
Q2G2Q4	0.030749	1.72	*rbfA*	Ribosome-binding factor A
Q2G0L1	0.031244	1.72	*folE2*	GTP cyclohydrolase FolE2 (EC 3.5.4.16)
Q2FXY0	0.021805	1.72	*SAOUHSC_01700*	CP-type G domain-containing protein
Q2FY89	0.001147	1.72	*SAOUHSC_01575*	Helix-turn-helix domain protein
Q2FUZ8	0.012386	1.71	*SAOUHSC_02947*	Sulfite reductase (NADPH) flavoprotein alpha-component, putative (EC 1.8.1.2)
Q2FXF8	0.007275	1.70	*SAOUHSC_01890*	NAD_binding_9 domain-containing protein
Q2FWH5	0.023599	1.69	*cshA*	DEAD-box ATP-dependent RNA helicase CshA (EC 3.6.4.13)

Q2G1G8	0.011674	1.69	*ptsG*	PTS system glucose-specific EIICBA component (EC 2.7.1.199)
Q2FXH0	0.018408	1.69	*SAOUHSC_01877*	Radical SAM core domain-containing protein
Q2FWH3	0.002414	1.68	*ddl*	D-alanine--D-alanine ligase (EC 6.3.2.4) (D-Ala-D-Ala ligase) (D-alanylalanine synthetase)
Q2FV10	0.01125	1.67	*SAOUHSC_02933*	Betaine aldehyde dehydrogenase (EC 1.2.1.8)
Q2G2L0	0.003802	1.66	*SAOUHSC_00638*	HTH dtxR-type domain-containing protein
Q2FVF9	0.029353	1.66	*SAOUHSC_02755*	Uncharacterized protein
Q2FXL1	0.046515	1.66	*thiI*	Probable tRNA sulfurtransferase (EC 2.8.1.4)
O50581	0.019771	1.65	*recG*	ATP-dependent DNA helicase RecG (EC 3.6.4.12)
gene_2675	0.003652	1.64	gene_2675	Uncharacterized protein
Q2G2N4	0.007706	1.63	*glcT*	Protein GlcT
Q2FVT6	0.006631	1.63	*hutI*	Imidazolonepropionase (EC 3.5.2.7) (Imidazolone-5-propionate hydrolase)
Q2FVI6	0.004699	1.62	*SAOUHSC_02724*	Uncharacterized protein
Q2G026	0.007622	1.61	*SAOUHSC_00801*	Protein translocase subunit SecG
Q2G242	0.029164	1.61	*SAOUHSC_00508*	TRAM domain-containing protein
Q2FWD8	0.029487	1.61	*rpmE2*	50S ribosomal protein L31 type B
Q2FYI6	0.035684	1.61	*SAOUHSC_01460*	THUMP domain-containing protein
Q2G0P7	0.033224	1.60	*mcsA*	Protein-arginine kinase activator protein
Q2G2A5	0.001039	1.60	*SAOUHSC_01041*	Pyruvate dehydrogenase complex, E1 component, pyruvate dehydrogenase beta subunit, putative (EC 1.2.4.1)
Q2G0M9	0.033549	1.60	*SAOUHSC_00531*	M20_dimer domain-containing protein
Q9RFJ6	0.00431	1.59	*rot*	HTH-type transcriptional regulator rot (Repressor of toxins)
Q2G261	0.026092	1.58	*sodM*	Superoxide dismutase [Mn/Fe] 2 (EC 1.15.1.1)
Q2FZV1	0.048026	1.57	*mnhA1*	Na(+)/H(+) antiporter subunit A1 (Mnh complex subunit A1)
Q2FXP2	0.006026	1.56	*SAOUHSC_01794*	Glyceraldehyde-3-phosphate dehydrogenase (EC 1.2.1.-)
Q2FWE7	0.013914	1.56	*atpH*	ATP synthase subunit delta (ATP synthase F(1) sector subunit delta) (F-type ATPase subunit delta)
Q2FYQ2	0.006605	1.56	*SAOUHSC_01383*	Uncharacterized protein
Q2G241	0.002752	1.55	*gltX*	Glutamate--tRNA ligase (EC 6.1.1.17) (Glutamyl-tRNA synthetase) (GluRS)
Q7BHL7	0.019059	1.55	*msrR*	Regulatory protein MsrR
Q2FWW9	0.047203	1.55	*SAOUHSC_02152*	ABC transporter, ATP-binding protein, putative
Q2G0R8	0.005176	1.55	*mfd*	Transcription-repair-coupling factor (TRCF) (EC 3.6.4.-)
Q2FVQ1	0.040466	1.55	*SAOUHSC_02651*	N-acetyltransferase domain-containing protein
Q2FV11	0.003528	1.54	*betA*	Oxygen-dependent choline dehydrogenase (CDH) (CHD) (EC 1.1.99.1) (Betaine aldehyde dehydrogenase) (BADH)

Q2G0I8	0.028985	1.54	*SAOUHSC_00577*	Mevalonate kinase (EC 2.7.1.36)
Q2FZ77	0.023331	1.54	*pyrR*	Bifunctional protein PyrR [Includes: Pyrimidine operon regulatory protein; Uracil phosphoribosyltransferase
Q2FXG2	0.022913	1.54	*ribH*	6,7-dimethyl-8-ribityllumazine synthase (DMRL synthase) (LS) (Lumazine synthase) (EC 2.5.1.78)
Q2G2F1	0.01137	1.54	*SAOUHSC_01967*	ABC transporter, ATP-binding protein, putative
Q2FV24	0.008504	1.53	*SAOUHSC_02915*	PepX_C domain-containing protein
Q2G2Q2	0.010743	1.53	*SAOUHSC_01249*	Riboflavin biosynthesis protein [Includes: Riboflavin kinase (EC 2.7.1.26)
Q2FXX8	0.016066	1.52	*mtnN*	5'-methylthioadenosine/S-adenosylhomocysteine nucleosidase (MTA/SAH nucleosidase) (EC 3.2.2.9)
Q2FX00	0.041842	1.52	*gatD*	Lipid II isoglutaminyl synthase (glutamine-hydrolyzing) subunit GatD (EC 6.3.5.13)
Q2FXN6	0.003779	1.52	*SAOUHSC_01800*	Two-component response regulator, putative
Q2FW11	0.001482	1.52	*rplV*	50S ribosomal protein L22
Q2FYJ1	0.027013	1.51	*SAOUHSC_01454*	53EXOc domain-containing protein
Q2FUQ2	0.002351	1.51	*mnmE*	tRNA modification GTPase MnmE (EC 3.6.-.-)
gene_4	0.004236	1.50	gene_4	Uncharacterized protein
Q2G015	0.000851	0.66	*clfA*	Clumping factor A (Fibrinogen receptor A) (Fibrinogen-binding protein A)
Q2FZ29	0.027364	0.65	*hslV*	ATP-dependent protease subunit HslV (EC 3.4.25.2)
Q2FWX6	0.043538	0.65	*SAOUHSC_02145*	Uncharacterized protein
Q2FXV1	0.041905	0.65	*SAOUHSC_01730*	UPF0337 protein SAOUHSC_01730
Q2FUY9	0.032002	0.65	*SAOUHSC_02956*	DNA-binding response regulator, putative
Q2FWE5	0.019132	0.65	*glyA*	Serine hydroxymethyltransferase (SHMT) (Serine methylase) (EC 2.1.2.1)
Q2G0F3	0.009223	0.65	*SAOUHSC_00616*	AB hydrolase-1 domain-containing protein
Q2FZL1	0.005598	0.64	*SAOUHSC_00989*	Aminotransferase (EC 2.6.1.-)
Q2FVR9	0.045179	0.64	*fni*	Isopentenyl-diphosphate delta-isomerase (IPP isomerase) (EC 5.3.3.2)
Q2FW16	0.007272	0.63	*rplN*	50S ribosomal protein L14
Q2G0V0	0.010265	0.62	*SAOUHSC_00426*	Lipoprotein
Q2FYM3	0.017205	0.62	*SAOUHSC_01415*	VOC domain-containing protein
Q2FZI9	0.035066	0.62	*purF*	Amidophosphoribosyltransferase (ATase) (EC 2.4.2.14)
Q2FYK5	0.037341	0.60	*thyA*	Thymidylate synthase (TS) (TSase) (EC 2.1.1.45)
Q2G122	0.02241	0.60	*metE*	5-methyltetrahydropteroyltriglutamate--homocysteine methyltransferase (EC 2.1.1.14)
gene_2722	0.00095	0.60	gene_2722	Uncharacterized protein
Q2FZI0	0.019489	0.60	*SAOUHSC_01024*	Uncharacterized protein
Q2FZJ2	0.000794	0.60	*purS*	Phosphoribosylformylglycinamidine synthase subunit PurS (FGAM synthase) (EC 6.3.5.3)
Q2FV63	0.043092	0.60	*copZ*	Copper chaperone CopZ

Q2FY42	0.01403	0.59	*SAOUHSC_01624*	Biotin carboxyl carrier protein of acetyl-CoA carboxylase
Q2FZ13	0.012868	0.59	*SAOUHSC_01258*	DUF3388 domain-containing protein
Q2FVK1	0.013853	0.59	*hlgB*	Gamma-hemolysin component B (H-gamma-1) (H-gamma-I) (Leukocidin f subunit)
Q2FZG5	0.00169	0.59	*SAOUHSC_01039*	Uncharacterized protein
Q2FYM8	0.011446	0.59	*SAOUHSC_01407*	Uncharacterized protein
Q2FVJ7	0.00983	0.59	*bioB*	Biotin synthase (EC 2.8.1.6)
Q2G2X2	0.019236	0.59	*tarD*	Glycerol-3-phosphate cytidylyltransferase (GCT) (GCTase) (Gro-PCT) (EC 2.7.7.39)
Q2FWJ3	0.010644	0.59	*rsbW*	Serine-protein kinase RsbW (EC 2.7.11.1) (Anti-sigma-B factor) (Sigma-B negative effector RsbW)
gene_2640	0.026994	0.58	gene_2640	Uncharacterized protein
gene_1727	0.002046	0.58	gene_1727	Uncharacterized protein
Q2G2F0	0.006898	0.57	*SAOUHSC_01968*	HIT domain-containing protein
Q2FVV1	0.037298	0.57	*SAOUHSC_02590*	AA_permease domain-containing protein
Q2G2H1	0.01417	0.57	*SAOUHSC_02014*	Uncharacterized protein
Q2FV87	0.011435	0.57	*glcB*	PTS system glucoside-specific EIICBA component
Q2FVT5	0.003043	0.55	*hutU*	Urocanate hydratase (Urocanase) (EC 4.2.1.49) (Imidazolonepropionate hydrolase)
Q2FXD3	0.034173	0.55	*SAOUHSC_01930*	Uncharacterized protein
Q2G0Z9	0.037058	0.55	*SAOUHSC_00362*	Uncharacterized protein
Q2FVL7	0.003644	0.54	*SAOUHSC_02694*	Thioredoxin-like_fold domain-containing protein
Q2G0F2	0.00333	0.54	*SAOUHSC_00617*	Uncharacterized protein
Q2FZR6	0.010204	0.52	*SAOUHSC_00924*	ABC transmembrane type-1 domain-containing protein
Q2FVZ7	0.002139	0.52	*SAOUHSC_02523*	Uncharacterized protein
P60070	0.013321	0.52	*rsbV*	Anti-sigma-B factor antagonist (Anti-anti-sigma-B factor)
Q2G229	0.007995	0.51	*SAOUHSC_00452*	Uncharacterized protein
Q2G189	0.03868	0.51	*esxA*	Type VII secretion system extracellular protein A (Ess extracellular protein A)
Q2FXW2	0.00394	0.51	*SAOUHSC_01719*	UPF0473 protein SAOUHSC_01719
Q2G2P8	0.016525	0.50	*nnrD*	ADP-dependent (S)-NAD(P)H-hydrate dehydratase (EC 4.2.1.136)
Q2FY07	0.024184	0.49	*recO*	DNA repair protein RecO (Recombination protein O)
Q2FUY2	0.003116	0.48	*clfB*	Clumping factor B (Fibrinogen receptor B) (Fibrinogen-binding protein B)
Q2FZK7	0.0043	0.48	*atl*	Bifunctional autolysin [Includes: N-acetylmuramoyl-L-alanine amidase (EC 3.5.1.28)
Q2G105	0.002137	0.48	*SAOUHSC_00356*	Uncharacterized protein
Q2FWQ5	0.017674	0.47	*SAOUHSC_02225*	Conserved hypothetical phage protein
Q2FXV2	0.007817	0.47	*SAOUHSC_01729*	Uncharacterized protein
Q2G2P5	0.009587	0.45	*nikA*	Nickel-binding protein NikA (SaNikA)
Q2FWP0	0.005973	0.44	*SAOUHSC_02241*	Uncharacterized leukocidin-like protein 1

Q2FZR3	0.006434	0.44	*SAOUHSC_00927*	Oligopeptide ABC transporter, substrate-binding protein, putative
Q2G0L4	4.19E-05	0.44	*sdrD*	Serine-aspartate repeat-containing protein D
Q2FVE7	0.008769	0.43	*cntA*	Metal-staphylopine-binding protein CntA
Q2FVK5	0.00112	0.43	*sbi*	Immunoglobulin-binding protein Sbi
Q2G160	0.007741	0.40	*nanA*	N-acetylneuraminate lyase (NAL) (Neu5Ac lyase) (EC 4.1.3.3)
Q2FY22	0.018063	0.40	*rpmG2*	50S ribosomal protein L33 2
Q2G1T5	0.000774	0.40	*SAOUHSC_02802*	Fibronectin binding protein B, putative
Q2FWQ7	0.006135	0.40	*SAOUHSC_02223*	Phi PVL orf 39-like protein
Q2G253	0.014238	0.38	*SAOUHSC_00025*	GRAM_POS_ANCHORING domain-containing protein
Q2FYL4	0.028796	0.37	*SAOUHSC_01425*	N-acetyltransferase domain-containing protein
Q2FV42	0.013443	0.37	*SAOUHSC_02897*	HTH-type transcriptional regulator SAOUHSC_02897
Q2G188	0.027257	0.37	*esaA*	Type VII secretion system accessory factor EsaA
Q2FWN9	0.014951	0.34	*SAOUHSC_02243*	Uncharacterized leukocidin-like protein 2
Q2FWU1	0.011002	0.34	*SAOUHSC_02184*	Phi PVL orf 14-like protein
Q2FXT2	0.006787	0.33	*SAOUHSC_01752*	UPF0735 ACT domain-containing protein SAOUHSC_01752
Q2G0B5	0.000295	0.32	*SAOUHSC_00690*	Uncharacterized protein
Q2FZU3	0.009435	0.30	*SAOUHSC_00897*	GP-PDE domain-containing protein
gene_1146	0.007575	0.28	gene_1146	Uncharacterized protein
Q2FZS2	0.005342	0.28	*SAOUHSC_00918*	Truncated MHC class II analog protein
Q2G037	0.006948	0.23	*whiA*	Probable cell division protein WhiA
Q2G1A4	-	(-3)	*SAOUHSC_00241*	Uncharacterized protein
Q2FYW7	-	(-3.33)	*SAOUHSC_01306*	Uncharacterized protein
gene_1967	-	(-3.66)	*gene_1967*	Uncharacterized protein
gene_2700	-	(-4.33)	*gene_2700*	Uncharacterized protein
Q9ZNI1	-	(-4.33)	*lytN*	Probable cell wall hydrolase LytN (EC 3.-.-.-)
gene_1145	-	(-4.66)	*gene_1145*	Uncharacterized protein
Q2G0A1	-	(-5)	*SAOUHSC_00704*	Uncharacterized protein
Q2FZY9	-	(-6)	*SAOUHSC_00845*	UPF0337 protein SAOUHSC_00845
Q2G1X0	-	(-6.33)	*hly*	Alpha-hemolysin (Alpha-HL) (Alpha-toxin)
Q2G0X2	-	(-6.33)	*SAOUHSC_00401*	Uncharacterized protein
Q2G010	-	(-6.66)	*SAOUHSC_00818*	Micrococcal nuclease (EC 3.1.31.1) (Staphylococcal nuclease) (Thermonuclease)
Q2FXH4	-	(-7.66)	*SAOUHSC_01873*	Uncharacterized protein

Table S6. List of the differentially expressed proteins in the *yycH* tolerant population upon daptomycin treatment compared to the untreated one.

Protein	*p-value*	Fold change / (average PSM)	Gene	Protein
Q2FWU3	-	(23)	*SAOUHSC_02182*	Lysostaphin (EC 3.4.24.75)
Q2G1F1	-	(12)	*SAOUHSC_00174*	Lysostaphin (EC 3.4.24.75)
Q2FY44	-	(10.33)	*SAOUHSC_01622*	Alkaline shock protein 23
Q2FY74	-	(10)	*xerD*	Tyrosine recombinase XerD
Q2G0V3	-	(10)	*SAOUHSC_00422*	Trans-sulfuration enzyme family protein, putative
gene_2767	-	(9.66)	gene_2767	Uncharacterized protein
Q2G2K0	-	(9.33)	*SAOUHSC_01530*	Conserved hypothetical phage protein
Q2FXU8	-	(9)	*SAOUHSC_01734*	AAA domain-containing protein
Q2G238	-	(7.66)	*SAOUHSC_00707*	Tagatose-6-phosphate kinase (EC 2.7.1.144)
Q2FV43	-	(7.33)	*SAOUHSC_02896*	Uncharacterized protein
Q2FVY3	-	(7)	*SAOUHSC_02538*	Molybdopterin synthase sulfur carrier subunit
Q2FWT7	-	(6.66)	*SAOUHSC_02188*	Phage head-tail adaptor, putative
Q2FWU4	-	(6.66)	*SAOUHSC_02181*	Phi PVL orfs 18-19-like protein
Q2FWV3	-	(6.33)	*SAOUHSC_02171*	Staphylokinase
Q2FV61	-	(6)	*SAOUHSC_02876*	Aminotransferase (EC 2.6.1.-)
Q2FXQ9	-	(6)	*hemA*	Glutamyl-tRNA reductase (GluTR) (EC 1.2.1.70)
Q2FZQ1	-	(6)	*SAOUHSC_00949*	Uncharacterized protein
Q2FVP9	-	(5.66)	*SAOUHSC_02653*	GCN5-related N-acetyltransferase
Q2G0E8	-	(5.66)	*SAOUHSC_00658*	Phosphoenolpyruvate--glycerone phosphotransferase (EC 2.7.1.121)
Q2FXI2	-	(5.66)	*trmB*	tRNA (guanine-N(7)-)-methyltransferase (EC 2.1.1.33)
Q2G134	-	(5.33)	*SAOUHSC_00322*	Uncharacterized protein
Q2FZ56	-	(5)	*fapR*	Transcription factor FapR (Fatty acid and phospholipid biosynthesis regulator)
Q2FWN2	-	(4.66)	*SAOUHSC_02256*	Abortive infection protein
Q2G148	-	(4.66)	*SAOUHSC_00307*	Deacetylase sirtuin-type domain-containing protein
Q2FV65	-	(4.66)	*SAOUHSC_02872*	Uncharacterized protein
Q2FZL6	-	(4.66)	*menH*	Putative 2-succinyl-6-hydroxy-2,4-cyclohexadiene-1-carboxylate synthase (SHCHC synthase) (EC 4.2.99.20)
Q2G0Y5	-	(4.33)	*SAOUHSC_00376*	Uncharacterized protein
Q2G2X5	-	(4.33)	*SAOUHSC_00642*	Transport permease protein
gene_2690	-	(4.33)	gene_2690	Uncharacterized protein
Q2G144	-	(4.33)	*SAOUHSC_00311*	PTS EIIB type-2 domain-containing protein
Q2FXW9	-	(4)	*SAOUHSC_01711*	AHS2 domain-containing protein
Q2G2S1	-	(4)	*SAOUHSC_01536*	ATP-dependent Clp protease proteolytic subunit
Q2FV26	-	(4)	*SAOUHSC_02913*	HTH tetR-type domain-containing protein
Q2FZT8	-	(4)	*SAOUHSC_00902*	Signal peptidase I (EC 3.4.21.89)

Q2FUS8	-	(3.66)	*drp35*	Lactonase drp35 (EC 3.1.1.-)
Q2G0U4	-	(3.66)	*SAOUHSC_00433*	Uncharacterized protein
Q2FYN7	-	(3.66)	*dapH*	2,3,4,5-tetrahydropyridine-2,6-dicarboxylate N-acetyltransferase (EC 2.3.1.89)
Q2FV94	-	(3.66)	*SAOUHSC_02840*	L-serine deaminase
Q2G0U8	-	(3.66)	*SAOUHSC_00428*	Uncharacterized protein
Q2FW95	-	(3.66)	*SAOUHSC_02404*	Uncharacterized protein
Q2G097	-	(3.66)	*SAOUHSC_00724*	Chorismate binding enzyme, putative
Q2G1N2	-	(3.33)	*sbnB*	N-((2S)-2-amino-2-carboxyethyl)-L-glutamate dehydrogenase (EC 1.5.1.51)
P69848	-	(3.33)	*moaA*	GTP 3',8-cyclase (EC 4.1.99.22) (Molybdenum cofactor biosynthesis protein A)
Q2FX06	-	(3.33)	*SAOUHSC_02101*	Uncharacterized protein
Q2FZX7	-	(3.33)	*SAOUHSC_00858*	Uncharacterized protein
Q2G1I3	-	(3.33)	*SAOUHSC_00139*	Uncharacterized protein
Q2G090	-	(3.33)	*SAOUHSC_00730*	DNA helicase (EC 3.6.4.12)
Q2G2V4	-	(3.33)	*SAOUHSC_01914*	Putative membrane protein insertion efficiency factor
Q2FVB9	-	(3.33)	*SAOUHSC_02815*	MFS domain-containing protein
Q2G1A8	-	(3)	*SAOUHSC_00237*	Methyltransf_25 domain-containing protein
Q2FV69	-	(3)	*SAOUHSC_02867*	HTH tetR-type domain-containing protein
Q2FYR8	-	(3)	*SAOUHSC_01367*	Anthranilate synthase component II, putative
Q2FWU5	0.009889	12.44	*SAOUHSC_02180*	Phage minor structural protein, N-terminal region domain protein
Q2FVQ4	0.012187	7.11	*SAOUHSC_02648*	L-lactate permease
Q2FZ30	0.003427	6.86	*xerC*	Tyrosine recombinase XerC
Q2FZX3	2.12E-05	5.04	*SAOUHSC_00862*	Uncharacterized protein
Q2G0L7	0.000588	4.25	*azo1*	FMN-dependent NADPH-azoreductase (EC 1.7.-.-)
Q2G0T1	0.015274	4.20	*rnmV*	Ribonuclease M5 (EC 3.1.26.8) (RNase M5) (Ribosomal RNA terminal maturase M5)
Q2FYY8	0.006584	4.12	*SAOUHSC_01284*	Uncharacterized protein
Q2G1U2	0.004094	3.71	*SAOUHSC_01174*	3-dmu-9_3-mt domain-containing protein
Q2G0E7	0.007145	3.60	*SAOUHSC_00659*	Uncharacterized protein
Q2FX98	0.004072	3.29	*SAOUHSC_01979*	HTH cro/C1-type domain-containing protein
Q2FV44	0.003412	3.13	*SAOUHSC_02895*	NmrA domain-containing protein
Q2FW01	0.047754	3.02	*SAOUHSC_02519*	N-acetyltransferase domain-containing protein
Q2G2U5	0.033054	3.02	*SAOUHSC_00024*	Lactamase_B domain-containing protein
Q2G2K5	0.012455	2.88	*ureC*	Urease subunit alpha (EC 3.5.1.5) (Urea amidohydrolase subunit alpha)
Q2FXW4	0.026723	2.84	*SAOUHSC_01717*	Uncharacterized protein
Q2FYP0	0.00756	2.83	*asd*	Aspartate-semialdehyde dehydrogenase (ASA dehydrogenase) (ASADH) (EC 1.2.1.11)
Q2G159	0.00083	2.75	*SAOUHSC_00296*	ROK family protein
Q2G084	0.013952	2.73	*SAOUHSC_00736*	Putative lipid kinase SAOUHSC_00736 (EC 2.7.1.-)
Q2G0A0	0.035442	2.71	*SAOUHSC_00705*	Cys-tRNA(Pro)/Cys-tRNA(Cys) deacylase (EC 4.2.-.-)

Q2FXW3	0.01879	2.67	*trmR*	tRNA 5-hydroxyuridine methyltransferase (EC 2.1.1.-) (ho5U methyltransferase)
Q2FXZ6	0.02836	2.65	*SAOUHSC_01679*	Uncharacterized protein
Q2FWG8	0.01343	2.64	*SAOUHSC_02323*	Cardiolipin synthase (CL synthase) (EC 2.7.8.-)
Q2FVJ5	0.033265	2.62	*bioD*	ATP-dependent dethiobiotin synthetase BioD (EC 6.3.3.3) (DTB synthetase) (DTBS) (Dethiobiotin synthase)
Q2FW06	0.042728	2.51	*rplC*	50S ribosomal protein L3
Q2FZH1	0.037175	2.47	*SAOUHSC_01034*	Uncharacterized protein
Q2FW56	0.011986	2.46	*SAOUHSC_02461*	Transcriptional regulator, merR family, putative
Q2FX00	0.025493	2.40	*gatD*	Lipid II isoglutaminyl synthase (glutamine-hydrolyzing) subunit GatD (EC 6.3.5.13)
Q2FZ87	0.045002	2.37	*SAOUHSC_01153*	Pyridoxal phosphate homeostasis protein (PLP homeostasis protein)
gene_2653	0.024264	2.30	*gene_2653*	Uncharacterized protein
Q2FY13	0.039039	2.29	*SAOUHSC_01661*	Uncharacterized protein
Q93Q23	0.008062	2.28	*mgt*	Monofunctional glycosyltransferase (MGT) (EC 2.4.1.129) (Peptidoglycan TGase)
Q2G0E1	0.00823	2.28	*graX*	Auxiliary protein GraX
O33599	0.01888	2.25	*lytM*	Glycyl-glycine endopeptidase LytM (EC 3.4.24.75) (Autolysin LytM)
Q2FZP4	0.046919	2.24	*prfC*	Peptide chain release factor 3 (RF-3)
Q2G1U9	0.017599	2.22	*SAOUHSC_02689*	Uncharacterized protein
Q2G1N4	0.044473	2.21	*SAOUHSC_00074*	Periplasmic binding protein, putative
Q2FY55	0.013329	2.21	*SAOUHSC_01610*	UPF0403 protein SAOUHSC_01610
Q2FZ05	0.035926	2.20	*SAOUHSC_01266*	Uncharacterized protein
Q2FXI7	0.011004	2.19	*SAOUHSC_01859*	UPF0354 protein SAOUHSC_01859
Q2FWF8	0.001097	2.12	*sceD*	Probable transglycosylase SceD (EC 3.2.-.-)
Q2FYN6	0.024259	2.12	*SAOUHSC_01399*	Uncharacterized hydrolase SAOUHSC_01399 (EC 3.-.-.-)
Q2G0D8	0.007294	2.08	*SAOUHSC_00667*	Putative hemin import ATP-binding protein HrtA
P0A0K3	0.010317	2.07	*SAOUHSC_02013*	Uncharacterized protein SAOUHSC_02013 (ORF1)
Q2FW72	0.007715	2.05	*sfnaB*	Staphyloferrin A synthase (EC 6.3.2.57)
Q2FXX2	0.032042	2.04	*pxpA*	5-oxoprolinase subunit A (5-OPase subunit A) (EC 3.5.2.9) (5-oxoprolinase (ATP-hydrolyzing) subunit A)
Q2G065	0.013755	2.00	*SAOUHSC_00756*	Uncharacterized protein
gene_4	0.03312	1.97	*gene_4*	Uncharacterized protein
Q2G2D5	0.047762	1.97	*lacG*	6-phospho-beta-galactosidase (EC 3.2.1.85) (Beta-D-phosphogalactoside galactohydrolase)
Q2G1I7	0.024641	1.95	*SAOUHSC_00135*	Uncharacterized protein
Q2FYN2	0.001482	1.94	*cspA*	Cold shock protein CspA
Q2FY14	0.029369	1.93	*SAOUHSC_01660*	GTP cyclohydrolase 1 type 2 homolog
Q2G031	0.010528	1.93	*pgk*	Phosphoglycerate kinase (EC 2.7.2.3)
Q2FXJ9	0.045131	1.92	*SAOUHSC_01835*	Uncharacterized protein

Q2G298	0.000658	1.91	*rsfS*	Ribosomal silencing factor RsfS
Q2FYU2	0.032151	1.88	*SAOUHSC_01332*	Uncharacterized protein
Q2FVW3	0.023296	1.87	*SAOUHSC_02579*	FAD_binding_3 domain-containing protein
Q2G2W9	0.010076	1.85	*SAOUHSC_00455*	PSP1 C-terminal domain-containing protein
Q2FVP8	0.009216	1.84	*SAOUHSC_02654*	Ferredoxin--NADP reductase (FNR) (Fd-NADP(+) reductase) (EC 1.18.1.2)
Q2FY10	0.011096	1.84	*SAOUHSC_01664*	Putative pyruvate, phosphate dikinase regulatory protein (PPDK regulatory protein)
Q2FVH5	0.037891	1.81	*SAOUHSC_02737*	Epimerase/dehydratase, putative
Q2FXI5	0.032134	1.80	*SAOUHSC_01861*	Uncharacterized protein
Q2FX14	0.039319	1.75	*SAOUHSC_02092*	Aminopeptidase PepS, putative (EC 3.4.11.-)
Q2G0R6	0.008308	1.73	*SAOUHSC_00480*	Uncharacterized protein
Q2FVJ7	0.014272	1.72	*bioB*	Biotin synthase (EC 2.8.1.6)
Q2FY27	0.003401	1.67	*SAOUHSC_01646*	Glucokinase (EC 2.7.1.2) (Glucose kinase)
Q2G066	0.041888	1.67	*SAOUHSC_00755*	Uncharacterized protein
Q2G029	0.001401	1.65	*gpmI*	2,3-bisphosphoglycerate-independent phosphoglycerate mutase (BPG-independent PGAM) (EC 5.4.2.12)
Q2FWM9	0.013072	1.64	*SAOUHSC_02259*	CN hydrolase domain-containing protein
Q2G0U0	0.023302	1.62	*SAOUHSC_00437*	Uncharacterized protein
Q2FZ53	0.016958	1.60	*SAOUHSC_01199*	3-oxoacyl-[acyl-carrier-protein] reductase (EC 1.1.1.100)
Q2FXX0	0.032785	1.59	*SAOUHSC_01710*	Acetyl-CoA carboxylase, biotin carboxyl carrier protein, putative
Q2FUS9	0.045994	1.57	*SAOUHSC_03022*	UPF0312 protein SAOUHSC_03022
Q2FZC4	0.028105	1.57	*SAOUHSC_01108*	Phosphoesterase (EC 3.1.4.-)
Q2FZS3	0.039773	1.56	*SAOUHSC_00917*	Uncharacterized protein
Q2FW21	0.031448	1.55	*rplF*	50S ribosomal protein L6
Q2FY34	0.00611	1.54	*gcvPA*	Probable glycine dehydrogenase (decarboxylating) subunit 1 (EC 1.4.4.2)
Q2FXY6	0.043021	1.52	*rpsT*	30S ribosomal protein S20
Q2FZW3	0.011872	1.51	*dltD*	Protein DltD
Q2G025	0.048966	1.51	*SAOUHSC_00802*	Carboxylesterase, putative (EC 3.1.1.1)
Q2G032	0.011068	1.51	*SAOUHSC_00795*	Glyceraldehyde-3-phosphate dehydrogenase (EC 1.2.1.-)
Q2FZ78	0.036529	1.50	*SAOUHSC_01163*	Pseudouridine synthase (EC 5.4.99.-)
Q2FXJ6	0.014016	1.50	*SAOUHSC_01838*	Serine protease HtrA-like
Q2FYM3	0.006346	0.66	*SAOUHSC_01415*	VOC domain-containing protein
Q2G1J4	0.038562	0.65	*SAOUHSC_00128*	Cap5O protein/UDP-N-acetyl-D-mannosaminuronic acid dehydrogenase (EC 1.1.1.-)
Q2G1Y6	0.019589	0.65	*bipA*	50S ribosomal subunit assembly factor BipA (EC 3.6.5.-) (GTP-binding protein BipA)
Q2FWH9	0.00901	0.65	*kdpA*	Potassium-transporting ATPase potassium-binding subunit
Q2G067	0.001859	0.64	*SAOUHSC_00754*	Uncharacterized protein
Q2G2W5	0.049116	0.64	*SAOUHSC_02630*	Biotin_lipoyl_2 domain-containing protein
Q2G0L4	0.00569	0.63	*sdrD*	Serine-aspartate repeat-containing protein D
P0A0H0	0.029028	0.63	*rpsL*	30S ribosomal protein S12

P72360	0.006948	0.63	*scdA*	Iron-sulfur cluster repair protein ScdA (Cell wall-related protein ScdA)
gene_2046	0.023265	0.63	*gene_2046*	Uncharacterized protein
Q2G2Q3	0.013675	0.62	*truB*	tRNA pseudouridine synthase B (EC 5.4.99.25)
Q2FUU5	0.004495	0.62	*lipA*	Lipase 1 (EC 3.1.1.3) (Glycerol ester hydrolase 1)
Q2G2C4	0.044229	0.61	*tarI'*	Ribitol-5-phosphate cytidylyltransferase 2 (EC 2.7.7.40)
Q2FUY2	0.000306	0.61	*clfB*	Clumping factor B (Fibrinogen receptor B) (Fibrinogen-binding protein B)
Q2FYJ1	0.007934	0.61	*SAOUHSC_01454*	53EXOc domain-containing protein
Q2FVS4	0.013466	0.60	*SAOUHSC_02618*	Uncharacterized protein
Q2G1T5	0.004292	0.60	*SAOUHSC_02802*	Fibronectin binding protein B, putative
Q2FWI1	0.012047	0.60	*kdpC*	Potassium-transporting ATPase KdpC subunit (ATP phosphohydrolase [potassium-transporting] C chain)
Q2G2F2	0.010366	0.60	*SAOUHSC_01966*	Uncharacterized protein
Q2FVI0	0.042873	0.59	*SAOUHSC_02731*	Na_H_Exchanger domain-containing protein
Q2FVZ7	0.016774	0.59	*SAOUHSC_02523*	Uncharacterized protein
Q2FUY6	0.049757	0.58	*SAOUHSC_02958*	Alkaline phosphatase III, putative
Q2G222	8.65E-06	0.58	*SAOUHSC_02979*	N-acetylmuramoyl-L-alanine amidase domain-containing protein SAOUHSC_02979
Q2FZX0	0.000824	0.57	*nagD*	Acid sugar phosphatase (EC 3.1.3.-)
Q2FWB8	0.000155	0.57	*deoD*	Purine nucleoside phosphorylase DeoD-type (PNP) (EC 2.4.2.1)
Q2FUQ2	0.041188	0.57	*mnmE*	tRNA modification GTPase MnmE (EC 3.6.-.-)
Q2G038	0.027953	0.57	*SAOUHSC_00788*	Gluconeogenesis factor
Q2FV08	0.045275	0.55	*SAOUHSC_02935*	HTH-type transcriptional regulator
Q2G0F2	0.00393	0.53	*SAOUHSC_00617*	Uncharacterized protein
Q2FXV2	0.001383	0.52	*SAOUHSC_01729*	Uncharacterized protein
Q2FXW2	0.030891	0.52	*SAOUHSC_01719*	UPF0473 protein SAOUHSC_01719
Q2FVE0	0.014684	0.51	*SAOUHSC_02774*	CMD domain-containing protein
Q2FXG0	0.044924	0.51	*SAOUHSC_01888*	Riboflavin synthase, alpha subunit (EC 2.5.1.9)
Q2FY78	0.003319	0.49	*SAOUHSC_01587*	Pseudouridine synthase (EC 5.4.99.-)
Q2FWI3	0.019477	0.46	*SAOUHSC_02308*	Uncharacterized protein
Q2G188	0.027543	0.44	*esaA*	Type VII secretion system accessory factor EsaA
Q2FZU3	0.003477	0.44	*SAOUHSC_00897*	GP-PDE domain-containing protein
Q2FWN9	0.01846	0.43	*SAOUHSC_02243*	Uncharacterized leukocidin-like protein 2
Q2G0U9	0.035361	0.42	*sle1*	N-acetylmuramoyl-L-alanine amidase sle1 (EC 3.5.1.28)
Q2FVK3	0.002223	0.38	*SAOUHSC_02708*	Gamma-hemolysin h-gamma-ii subunit, putative
Q2G2B2	0.017914	0.38	*sasG*	Surface protein G
Q2FZR5	0.001606	0.37	*SAOUHSC_00925*	ABC transporter domain-containing protein
Q2FVM1	0.004332	0.34	*SAOUHSC_02681*	Nitrate reductase (quinone) (EC 1.7.5.1)
Q2FWP0	0.018786	0.34	*SAOUHSC_02241*	Uncharacterized leukocidin-like protein 1

Q2FUX3	0.005748	0.32	*isaB*	Immunodominant staphylococcal antigen B
Q2G0X2	0.038737	0.32	*SAOUHSC_00401*	Uncharacterized protein
Q2FV27	0.029428	0.31	*SAOUHSC_02912*	3-dmu-9_3-mt domain-containing protein
Q2G1N9	0.018803	0.14	*SAOUHSC_00067*	L-lactate permease
Q2FYF0	-	(-3)	*SAOUHSC_01502*	ATP-dependent DNA helicase RecQ, putative (EC 3.6.1.-)
Q2G0Q2	-	(-3)	*SAOUHSC_00497*	HTH-type transcriptional regulator NorG
gene_1145	-	(-4.66)	*gene_1145*	Uncharacterized protein
Q2FXH4	-	(-7.33)	*SAOUHSC_01873*	Uncharacterized protein
Q2G1D3	-	(-7.66)	*SAOUHSC_00192*	Coagulase

Table S7. List of the differentially expressed proteins in the resistant population compared to the ancestral.

Protein	*p-value*	Fold change / (average PSM)	Gene	Protein
Q2G2K5	-	(10.33)	*ureC*	Urease subunit alpha (EC 3.5.1.5) (Urea amidohydrolase subunit alpha)
Q2G1K5	-	(9)	*SAOUHSC_00117*	Capsular polysaccharide biosynthesis protein Cap5D, putative
Q2G1I6	-	(6)	*SAOUHSC_00136*	ABC transporter domain-containing protein
Q2FZU2	-	(5.33)	*argH*	Argininosuccinate lyase (ASAL) (EC 4.3.2.1) (Arginosuccinase)
Q2FYR7	-	(4.33)	*trpD*	Anthranilate phosphoribosyltransferase (EC 2.4.2.18)
Q2G2N5	-	(4)	*SAOUHSC_01354*	Sodium:alanine symporter family protein, putative
Q2G1F1	-	(3.33)	*SAOUHSC_00174*	Lysostaphin (EC 3.4.24.75)
Q2FUV7	-	(3)	*SAOUHSC_02994*	Uncharacterized protein
Q2FUU2	-	(3)	*hisA*	1-(5-phosphoribosyl)-5-[(5-phosphoribosylamino)methylideneamino] imidazole-4-carboxamide isomerase
Q2FVS9	-	(3)	*SAOUHSC_02613*	MOSC domain-containing protein
Q2FV83	0.020243865	3.41	*SAOUHSC_02852*	HTH lysR-type domain-containing protein
Q2FXI3	0.015844766	2.81	*SAOUHSC_01864*	Lactamase_B domain-containing protein
Q2FUY6	0.03894321	2.74	*SAOUHSC_02958*	Alkaline phosphatase III, putative
Q2FVX7	0.037913007	2.58	*SAOUHSC_02545*	ThiF domain-containing protein
Q2G1V4	0.029532973	2.38	*SAOUHSC_00333*	ABC transporter, ATP-binding protein, putative
Q2FXL1	0.016558501	2.00	*thiI*	Probable tRNA sulfurtransferase (EC 2.8.1.4)
Q2FXZ5	0.023387972	1.94	*SAOUHSC_01680*	Ribosomal RNA small subunit methyltransferase E (EC 2.1.1.193)
Q2G2B2	0.047376338	1.83	*sasG*	Surface protein G
Q2G0B2	0.04949959	1.81	*SAOUHSC_00693*	Uncharacterized protein
Q7X2S2	0.011541526	1.74	*arcC2*	Carbamate kinase 2 (EC 2.7.2.2)

Q2FZK3	0.034846685	1.68	*fmtA*	Teichoic acid D-alanine hydrolase (EC 3.1.1.103) (Teichoic acid D-alanine esterase)
Q2G1F2	0.007504426	1.67	*azoR*	FMN-dependent NADH:quinone oxidoreductase (EC 1.6.5.-)
Q2FWW1	0.027713589	1.62	*SAOUHSC_02161*	MHC class II analog protein
Q2G232	0.026027978	1.59	*SAOUHSC_00450*	Orn/Lys/Arg decarboxylase, putative
Q2FXK4	0.004511913	1.58	*SAOUHSC_01830*	GP-PDE domain-containing protein
Q2G1Z0	0.017919374	1.55	*SAOUHSC_00655*	DhaK domain-containing protein
Q2FYL5	0.019595032	1.50	*murG*	UDP-N-acetylglucosamine--N-acetylmuramyl-(pentapeptide) pyrophosphoryl-undecaprenol N-acetylglucosamine transferase
Q2FW03	0.004948279	0.67	*topB*	DNA topoisomerase 3 (EC 5.6.2.1) (DNA topoisomerase III)
gene_1863	0.006105895	0.66	gene_1863	Uncharacterized protein
gene_10	0.009002779	0.65	gene_10	Uncharacterized protein
Q2FY55	0.028700841	0.64	*SAOUHSC_01610*	UPF0403 protein SAOUHSC_01610
gene_1727	0.000922307	0.63	gene_1727	Uncharacterized protein
Q2FZH2	0.002060535	0.63	*SAOUHSC_01032*	Cytochrome d ubiquinol oxidase, subunit II, putative (EC 1.10.3.-)
Q2G1C9	0.005562744	0.63	*SAOUHSC_00196*	Uncharacterized protein
Q2G1D8	0.008964261	0.62	*pflB*	Formate acetyltransferase (EC 2.3.1.54) (Pyruvate formate-lyase)
Q2FVJ7	0.039511792	0.62	*bioB*	Biotin synthase (EC 2.8.1.6)
Q2FZR4	0.01039334	0.58	*SAOUHSC_00926*	Oligopeptide ABC transporter, ATP-binding protein, putative
Q2FV42	0.023076994	0.58	*SAOUHSC_02897*	HTH-type transcriptional regulator SAOUHSC_02897
Q2FW37	0.047434511	0.56	*truA*	tRNA pseudouridine synthase A (EC 5.4.99.12)
Q2G2G1	0.041347625	0.55	*SAOUHSC_00716*	Uncharacterized protein
O33599	0.022954403	0.54	*lytM*	Glycyl-glycine endopeptidase LytM (EC 3.4.24.75) (Autolysin LytM)
Q2FYJ3	0.015732984	0.54	*tdcB*	L-threonine dehydratase catabolic TdcB (EC 4.3.1.19) (Threonine deaminase)
Q2FZK7	0.014723491	0.54	*atl*	Bifunctional autolysin [Includes: N-acetylmuramoyl-L-alanine amidase (EC 3.5.1.28)
Q2FW97	0.046002855	0.54	*SAOUHSC_02402*	EIIA (EIII) (Mannitol-specific phosphotransferase enzyme IIA component) (PTS system mannitol-specific EIIA component)
Q2G1G2	0.030223803	0.51	*SAOUHSC_00162*	Type I restriction enzyme R Protein (EC 3.1.21.3)
Q2FZV0	0.022309835	0.46	*SAOUHSC_00890*	Uncharacterized protein
Q2FX13	0.016071325	0.46	*SAOUHSC_02093*	UPF0435 protein SAOUHSC_02093
Q2G208	0.032954792	0.42	*SAOUHSC_02809*	Gluconate operon transcriptional repressor, putative
Q2FZG8	0.017670607	0.40	*rpoY*	DNA-directed RNA polymerase subunit epsilon (RNAP epsilon subunit) (EC 2.7.7.6)

Q2FZB0	0.001689728	0.36	*argF*	Ornithine carbamoyltransferase (OTCase) (EC 2.1.3.3)
Q2G163	0.010149043	0.35	*psuG*	Pseudouridine-5'-phosphate glycosidase (PsiMP glycosidase) (EC 4.2.1.70)
Q2FZH1	0.003684866	0.32	*SAOUHSC_01034*	Uncharacterized protein
Q2FYV3	0.004739626	0.29	*thrC*	Threonine synthase (EC 4.2.3.1)
gene_1931	0.020450854	0.27	gene_1931	Uncharacterized protein
Q2FZY9	0.036042523	0.26	*SAOUHSC_00845*	UPF0337 protein SAOUHSC_00845
gene_2767	0.037548434	0.23	gene_2767	Uncharacterized protein
Q2FVB9	0.029288378	0.23	*SAOUHSC_02815*	MFS domain-containing protein
Q2FZR6	0.005740066	0.22	*SAOUHSC_00924*	ABC transmembrane type-1 domain-containing protein
Q2G184	0.015789404	0.22	*essC*	Type VII secretion system protein EssC
Q2G138	0.000889858	0.13	*SAOUHSC_00317*	Glycerol-3-phosphate transporter
Q2FZQ1	-	(-3)	*SAOUHSC_00949*	Uncharacterized protein
Q2FX20	-	(-3)	*SAOUHSC_02086*	PV83 orf 4-like protein-related protein
Q2FY86	-	(-3.33)	*SAOUHSC_01578*	Conserved hypothetical phage protein
Q2FYW7	-	(-3.33)	*SAOUHSC_01306*	Uncharacterized protein
Q2FZZ2	-	(-3.66)	*metN2*	Methionine import ATP-binding protein MetN 2 (EC 7.4.2.11)
Q2G2H0	-	(-3.66)	*SAOUHSC_02010*	Uncharacterized protein
Q2FZ70	-	(-3.66)	*pyrE*	Orotate phosphoribosyltransferase (OPRT) (OPRTase) (EC 2.4.2.10)
Q2G0Y5	-	(-4)	*SAOUHSC_00376*	Uncharacterized protein
gene_1145	-	(-4.66)	*gene_1145*	Uncharacterized protein
Q2FV69	-	(-5)	*SAOUHSC_02867*	HTH tetR-type domain-containing protein
Q2FYF4	-	(-5.66)	*SAOUHSC_01497*	L-asparaginase, putative (EC 3.5.1.1)
Q2FZ30	-	(-5.66)	*xerC*	Tyrosine recombinase XerC
Q2FZA8	-	(-5.66)	*SAOUHSC_01130*	Uncharacterized protein
gene_2653	-	(-5.66)	*gene_2653*	Uncharacterized protein
Q2FZR5	-	(-6)	*SAOUHSC_00925*	ABC transporter domain-containing protein
Q2FV28	-	(-6)	*queH*	Epoxyqueuosine reductase QueH (EC 1.17.99.6) (Queuosine biosynthesis protein QueH)
Q2G1X0	-	(-6.33)	*hly*	Alpha-hemolysin (Alpha-HL) (Alpha-toxin)
Q2FYG6	-	(-7.66)	*SAOUHSC_01486*	Heptaprenyl diphosphate syntase component II, putative (EC 2.5.1.30)
Q2G2U2	-	(-10.33)	*yycI*	YycI domain-containing protein

Table S8. List of the differentially expressed proteins in the resistant population upon daptomycin treatment compared to the untreated one.

Protein	*p-value*	Fold change / (average PSM)	Gene	Protein
Q2FZ30	-	(22)	*xerC*	Tyrosine recombinase XerC
Q2FWU5	-	(19)	*SAOUHSC_02180*	Phage minor structural protein, N-terminal region domain protein
gene_2653	-	(15.33)	*gene_2653*	Uncharacterized protein
Q2FWU3	-	(15)	*SAOUHSC_02182*	Lysostaphin (EC 3.4.24.75)
Q2G2K0	-	(10.33)	*SAOUHSC_01530*	Conserved hypothetical phage protein
Q2G0V3	-	(10)	*SAOUHSC_00422*	Trans-sulfuration enzyme family protein, putative
Q2FW95	-	(10)	*SAOUHSC_02404*	Uncharacterized protein
Q2FUS7	-	(7.66)	*trhO*	tRNA uridine(34) hydroxylase (EC 1.14.-.-)
Q2FV28	-	(7.33)	*queH*	Epoxyqueuosine reductase QueH (EC 1.17.99.6)
Q2FWV3	-	(5.66)	*SAOUHSC_02171*	Staphylokinase
Q2G090	-	(5.66)	*SAOUHSC_00730*	DNA helicase (EC 3.6.4.12)
Q2FYG6	-	(5.66)	*SAOUHSC_01486*	Heptaprenyl diphosphate syntase component II, putative (EC 2.5.1.30)
Q2FWT7	-	(5.66)	*SAOUHSC_02188*	Phage head-tail adaptor, putative
Q2FXW9	-	(5)	*SAOUHSC_01711*	AHS2 domain-containing protein
Q2FZ56	-	(4.66)	*fapR*	Transcription factor FapR (Fatty acid and phospholipid biosynthesis regulator)
gene_1640	-	(4.33)	*gene_1640*	Uncharacterized protein
Q2G1N2	-	(4)	*sbnB*	N-((2S)-2-amino-2-carboxyethyl)-L-glutamate dehydrogenase (EC 1.5.1.51)
Q2FYF4	-	(4)	*SAOUHSC_01497*	L-asparaginase, putative (EC 3.5.1.1)
Q2FWX3	-	(3.66)	*SAOUHSC_02148*	Uncharacterized protein
Q2FZX7	-	(3.66)	*SAOUHSC_00858*	Uncharacterized protein
Q2FWU4	-	(3.33)	*SAOUHSC_02181*	Phi PVL orfs 18-19-like protein
Q2FYI1	-	(3.33)	*recU*	Holliday junction resolvase RecU (EC 3.1.22.4)
Q2G2H0	-	(3.33)	*SAOUHSC_02010*	Uncharacterized protein
Q2G0U8	-	(3.33)	*SAOUHSC_00428*	Uncharacterized protein
Q2FZ70	-	(3.33)	*pyrE*	Orotate phosphoribosyltransferase (OPRT) (OPRTase) (EC 2.4.2.10)
Q2FXZ0	-	(3.33)	*hrcA*	Heat-inducible transcription repressor HrcA
Q2FYR6	-	(3.33)	*trpC*	Indole-3-glycerol phosphate synthase (IGPS) (EC 4.1.1.48)
Q2G2L6	-	(3)	*SAOUHSC_02812*	Uncharacterized protein
Q2G1I3	-	(3)	*SAOUHSC_00139*	Uncharacterized protein
Q2FVD6	-	(3)	*SAOUHSC_02777*	Uncharacterized protein
Q2FVY3	-	(3)	*SAOUHSC_02538*	Molybdopterin synthase sulfur carrier subunit
gene_2	0.034971	8.15	*gene_2*	Uncharacterized protein
Q2FV43	0.031258	5.28	*SAOUHSC_02896*	Uncharacterized protein
O33599	0.007429	4.63	*lytM*	Glycyl-glycine endopeptidase LytM (EC 3.4.24.75) (Autolysin LytM)

gene_2709	0.032252	4.45	*gene_2709*	Uncharacterized protein
Q2FZH1	0.001247	4.17	*SAOUHSC_01034*	Uncharacterized protein
Q2FXQ9	0.012258	4.15	*hemA*	Glutamyl-tRNA reductase (GluTR) (EC 1.2.1.70)
Q2G1I7	0.002772	3.57	*SAOUHSC_00135*	Uncharacterized protein
Q2G0E7	0.002446	3.41	*SAOUHSC_00659*	Uncharacterized protein
Q2FX98	0.036464	3.34	*SAOUHSC_01979*	HTH cro/C1-type domain-containing protein
Q2FYV3	0.019575	3.28	*thrC*	Threonine synthase (EC 4.2.3.1)
Q2FWF8	0.012942	3.17	*sceD*	Probable transglycosylase SceD (EC 3.2.-.-)
Q93Q23	0.018049	2.73	*mgt*	Monofunctional glycosyltransferase (MGT) (EC 2.4.1.129) (Peptidoglycan TGase)
Q2FZS2	0.03752	2.59	*SAOUHSC_00918*	Truncated MHC class II analog protein
Q2FWU1	0.013507	2.57	*SAOUHSC_02184*	Phi PVL orf 14-like protein
Q2G0T9	0.033197	2.46	*SAOUHSC_00438*	Alpha amylase family protein, putative
Q2FYU2	0.000125	2.33	*SAOUHSC_01332*	Uncharacterized protein
Q2FVH5	0.003681	2.18	*SAOUHSC_02737*	Epimerase/dehydratase, putative
Q2FW37	0.030597	2.13	*truA*	tRNA pseudouridine synthase A (EC 5.4.99.12)
Q2FXX1	0.035883	1.95	*SAOUHSC_01709*	Acetyl-CoA carboxylase, biotin carboxylase, putative (EC 6.4.1.2)
Q2G122	0.030708	1.93	*metE*	5-methyltetrahydropteroyltriglutamate--homocysteine methyltransferase (EC 2.1.1.14)
Q2FV44	0.036769	1.93	*SAOUHSC_02895*	NmrA domain-containing protein
gene_4	0.008456	1.89	*gene_4*	Uncharacterized protein
Q2G298	0.031951	1.88	*rsfS*	Ribosomal silencing factor RsfS
Q2FXY3	0.025005	1.85	*nadD*	Probable nicotinate-nucleotide adenylyltransferase (EC 2.7.7.18)
Q2FZH2	0.001557	1.74	*SAOUHSC_01032*	Cytochrome d ubiquinol oxidase, subunit II, putative (EC 1.10.3.-)
Q2FVG8	0.025612	1.66	*SAOUHSC_02744*	Amino acid ABC transporter, ATP-binding protein, putative
Q2FZU7	0.006134	1.63	*SAOUHSC_00893*	FMN oxidoreductase, putative
Q2FZY3	0.024211	1.57	*SAOUHSC_00851*	UPF0051 protein SAOUHSC_00851
Q2G0G6	0.008139	1.55	*SAOUHSC_00603*	Aldo_ket_red domain-containing protein
Q2FVX3	0.021115	1.50	*fdhD*	Sulfur carrier protein FdhD
gene_2768	0.030441	0.66	*gene_2768*	Uncharacterized protein
Q2G297	0.049606	0.65	*SAOUHSC_01696*	HD domain-containing protein
Q2FZ91	0.006264	0.65	*divIB*	Cell division protein DivIB
Q2FXZ4	0.032557	0.64	*prmA*	Ribosomal protein L11 methyltransferase (L11 Mtase) (EC 2.1.1.-)
Q2G038	0.021145	0.59	*SAOUHSC_00788*	Gluconeogenesis factor
Q2FUX3	0.012921	0.57	*isaB*	Immunodominant staphylococcal antigen B
Q2FVH8	0.002011	0.57	*SAOUHSC_02733*	Membrane protein, putative
Q2FXI3	0.044005	0.55	*SAOUHSC_01864*	Lactamase_B domain-containing protein
Q2FVI3	0.025943	0.53	*SAOUHSC_02727*	Peptidase_C39_2 domain-containing protein
Q2FWP0	0.034254	0.53	*SAOUHSC_02241*	Uncharacterized leukocidin-like protein 1
Q2FWN9	0.045752	0.48	*SAOUHSC_02243*	Uncharacterized leukocidin-like protein 2

Q2G1X6	0.025947	0.47	*queC*	7-cyano-7-deazaguanine synthase (EC 6.3.4.20)
Q2G0B2	0.0111	0.47	*SAOUHSC_00693*	Uncharacterized protein
Q2G0X2	0.043407	0.40	*SAOUHSC_00401*	Uncharacterized protein
Q2FVM1	0.010696	0.25	*SAOUHSC_02681*	Nitrate reductase (quinone) (EC 1.7.5.1)
Q2G0U4	-	(-3)	*SAOUHSC_00433*	Uncharacterized protein
Q2FVS9	-	(-3)	*SAOUHSC_02613*	MOSC domain-containing protein
Q2FUU2	-	(-3)	*hisA*	1-(5-phosphoribosyl)-5-[(5-phosphoribosylamino)methylideneamino] imidazole-4-carboxamide isomerase
Q2G011	-	(-5.33)	*SAOUHSC_00817*	Uncharacterized protein
Q2G1U2	-	(-6)	*SAOUHSC_01174*	3-dmu-9_3-mt domain-containing protein
Q2G1I6	-	(-6)	*SAOUHSC_00136*	ABC transporter domain-containing protein
Q2G1K3	-	(-7.66)	*SAOUHSC_00119*	Capsular polysaccharide synthesis enzyme Cap8F
